# RAGE Re‐Expressed at Myofibre Level Drives Muscle Wasting in Cancer Conditions

**DOI:** 10.1002/jcsm.70302

**Published:** 2026-05-09

**Authors:** Sara Chiappalupi, Giulia Gentili, Laura Salvadori, Martina Paiella, Marcello Manfredi, Vittoria Federica Borrini, Kaamashri Mangar, Ann Marie Schmidt, Maurizio Muscaritoli, Francesca Riuzzi, Guglielmo Sorci

**Affiliations:** ^1^ Department of Medicine and Surgery University of Perugia Perugia Italy; ^2^ Interuniversity Institute of Myology (IIM) Perugia Italy; ^3^ Consorzio Interuniversitario Biotecnologie (CIB) Trieste Italy; ^4^ Department of Translational Medicine University of Piemonte Orientale Novara Italy; ^5^ Institute for Molecular and Translational Cardiology (IMTC), IRCCS Policlinico San Donato Milan Italy; ^6^ Department of Medicine NYU Grossman School of Medicine New York USA; ^7^ Department of Translational and Precision Medicine Sapienza University of Rome Rome Italy; ^8^ Centro Universitario di Ricerca sulla Genomica Funzionale (CURGeF) Perugia Italy

**Keywords:** animal models, cancer cachexia, muscle wasting, myofibre remodeling, RAGE

## Abstract

**Background:**

Cancer cachexia (CC) is a highly debilitating syndrome characterized by loss of body and muscle weight affecting most advanced cancer patients. The receptor for advanced glycation end‐products (RAGE) is expressed by several cell types and sustains the inflammatory response in acute and chronic diseases. Total ablation of RAGE (*Ager* ^−/−^ mice) translates into restrained CC and increased survival in tumour‐bearing mice. RAGE, which is not expressed in adult healthy myofibres, is re‐expressed in atrophying myofibres in cancer conditions. However, the specific contribution of muscular RAGE to CC was unknown.

**Methods:**

Using an HSA/Cre‐*loxP* system, we generated a tamoxifen‐inducible conditional *Ager* ^mKO^ mouse model in which RAGE is selectively ablated in myofibres. Tamoxifen‐treated *Ager* ^mKO^, *Ager* ^*flox*^ and *Ager* ^−/−^ mice were subcutaneously injected with Lewis lung carcinoma (LLC) cells, and body changes and survival were monitored until 25 dpi, when histological, molecular and proteomic analyses were performed in tumour‐bearing and control mice. Muscle samples of pre‐cachectic and cachectic pancreatic cancer patients were analysed to validate the results.

**Results:**

Compared with LLC‐*Ager* ^*flox*^ mice, LLC‐*Ager* ^mKO^ mice showed reduced (7.5% [*p =* 0.004] vs. 15.1% [*p* < 0.0001]) body weight loss, no significant reduction of hind‐limb muscle mass and strength and myofibre cross‐sectional areas, increased survival (69.2% vs. 42.9% mice alive at 25 dpi) and restrained muscle and serum pro‐inflammatory factors. Mechanistically, *Ager* ^mKO^ muscles resist cancer‐induced atrophy by maintaining an active Akt‐GSK‐3β‐PGC‐1α pathway, and increasing the synthesis of myosin heavy chain (MyHC)‐I and ‐IIa (71.8% [*p =* 0.008] and 73.9% [*p =* 0.002] increase, respectively) along with a 76.3% (*p =* 0.008) increase in hybrid MyHC‐I/IIa myofibres. Distinct proteomic signatures characterize muscles of tumour‐bearing mice in dependence on RAGE expression, supporting a protective effect of RAGE ablation in muscles. LLC/*Ager* ^mKO^ muscles showed increased amounts of several enzymes involved in glycolysis and glucose catabolism, typical of Warburg metabolism. Noteworthy, muscles of pre‐cachectic and cachectic cancer patients showed ~3‐fold increase (*p* < 0.05) in RAGE amounts and reduced Akt‐GSK‐3β‐PGC‐1α pathway, compared with healthy control subjects.

**Conclusions:**

Our data provide evidence that RAGE engagement at myofibre level drives loss of body and muscle weights and inflammation in cancer conditions. RAGE ablation in muscles confers resistance to CC through myofibre remodeling and glycolytic reprogramming. On the clinical side, the overexpression of RAGE is an early event in muscles of cancer patients, suggesting a role for RAGE in the onset of the cachectic syndrome. Thus, the molecular targeting of RAGE might be useful to counteract cachexia and prolong survival in cancer patients.

## 1 Introduction

Cancer cachexia (CC) is a disabling paraneoplastic syndrome characterized by body weight loss and muscle atrophy, leading to severe weakness and progressive functional impairment, reducing the tolerance and responsiveness to anticancer treatments and worsening the quality of life and survival of cancer patients [1]. Most malignant tumours (including pancreatic, pulmonary and colorectal cancers) culminate in CC, which has been estimated to be responsible for about half of all cancer deaths worldwide (i.e., ~8.2 million people per year), thus representing an urgent medical need and a major burden on the global healthcare system [1, 2]. CC is a multi‐organ syndrome, since its occurrence and development are closely related to changes in metabolic pathways induced by complex communications between the tumour cells and the host organs. Indeed, a plethora of CC factors (CCFs), such as inflammatory cytokines and circulating factors produced by cells of the tumour microenvironment, affect several host tissues, progressively increasing their catabolism. The immune system is first affected, representing a key driver for CC, ultimately leading to skeletal muscle wasting in the refractory stage [3]. Although some molecular mechanisms have been identified underlying CC, no effective therapy is currently available.

In the presence of cancer, circulating CCFs affect the physiological and dynamic balance between the contractile protein synthesis and degradation in muscles, leading to excessive catabolism. Proteolytic systems, principally the ubiquitin‐proteasome (UPS) and autophagy proteolytic (APS) systems, are hyperactivated by signaling pathways and transcription factors, while protein synthesis is deactivated [4]. In animal models of CC, the E3 ubiquitin‐protein ligases (also called atrogenes), tripartite motif containing 63 (*Trim63*; Murf1) and F‐box only protein 32 (*Fbxo32*; atrogin‐1), have a major role in the muscle catabolic process leading to selective degradation of contractile proteins, especially the fast isoforms of myosin heavy chain (MyHC), MyHC‐II [1, 2].

The receptor for advanced glycation end‐products (RAGE) is a member of the immunoglobulin superfamily of cell surface receptors. RAGE interacts with a plethora of ligands, including advanced glycation end‐products (AGEs) and damage‐associated molecular patterns (DAMPs), such as HMGB1 and S100 proteins. By activating multiple signaling pathways, RAGE exerts pleiotropic activities in physiological and pathological conditions, depending on the cell types and the presence and concentration of the specific ligands [5]. RAGE is expressed by several immune cell types and participates in innate and adaptive immune responses [6]. Moreover, RAGE sustains the inflammatory response in a plethora of acute and chronic diseases, mainly activating the transcription factor NF‐κB, which induces the expression of RAGE itself, thus generating an amplifying feedback loop [7, 8].

As occurs in most tissues, RAGE is highly expressed in skeletal muscles during development but is absent in healthy adult myofibres. However, RAGE is transiently re‐expressed in skeletal muscle tissue upon an acute injury to sustain the regeneration process [5, 9]. In several conditions, such as diabetes, ageing, obesity and certain myopathies, chronic expression of RAGE also occurs in myofibres, contributing to the muscle atrophy associated with these conditions [5].

Interestingly, a re‐expression of RAGE has been observed in atrophic myofibres of tumour‐bearing mice, along with high amounts of RAGE ligands released by tumour and inflammatory cells, potentially leading to a hyper‐stimulation of the receptor [10]. Data obtained in RAGE‐ablated mice highlight a main role of this receptor in sustaining CC. Indeed, tumour‐bearing RAGE‐null (*Ager* ^−/−^) mice showed a delayed onset of CC, reduced levels of CCFs and reduced loss of body weight and muscle mass and strength, translating into dramatically increased survival [10]. However, the specific contribution of RAGE expressed in the different tissue compartments and, in particular, in myofibres, in cancer conditions was still unknown.

Here, we evaluated the specific contribution of RAGE re‐expressed in the skeletal muscle compartment to the onset and progression of CC using a muscle‐specific conditional mouse model lacking RAGE only in skeletal myofibres (*Ager* ^mKO^ mice), in comparison with *Ager* ^−/−^ and control (*Ager* ^*flox*^) mice, and validated the results in muscle biopsies from pre‐cachectic and cachectic cancer patients.

## 2 Methods

### 2.1 Cell Culture

Lewis lung carcinoma (LLC) cells were cultured in high‐glucose Dulbecco's Modified Eagle's Medium (DMEM), supplemented with 10% heat‐inactivated foetal bovine serum (FBS), 100‐U/mL penicillin and 100‐μg/mL streptomycin. Cells were maintained in a humidified atmosphere containing 5% CO_2_ at 37°C.

### 2.2 Animal Models and In Vivo Experiments

C57BL/6 mice were obtained from Charles River Laboratories Italia. C57BL/6 *Ager* ^−/−^ mice [11] were originally obtained from Dr. Angelika Bierhaus (Heidelberg, Germany). C57BL/6 *Ager* ^*flox*^ mice carrying *loxP* ^+/+^ Cre^−/−^ (*loxP* sites flanking exons 4 and 7 of the *Ager* gene in both alleles) were engineered by A.M. Schmidt's laboratory [12]. C57BL/6 HSA‐MCM mice expressing MerCreMer (mutagen oestrogen receptor [Mer], double fusion protein with Cre recombinase) under the control of myofibre‐specific HSA promoter of the human *ACTA1* (actin α 1, skeletal muscle) [13] were from The Jackson Laboratory.

*Ager* ^mKO^ mice, in which the *Ager* gene is selectively deleted in myofibres in a tamoxifen‐inducible manner, were generated at Charles River Laboratories Italia. Female homozygous (*loxP* ^+/+^ Cre^−/−^) *Ager* ^*flox*^ mice were crossed with male hemizygous (*loxP* ^−/−^ Cre^+/−^) HSA‐MCM mice to obtain *loxP* ^+/−^ Cre^+/−^ mice in the F1 progeny. Female *loxP* ^+/+^ Cre^−/−^ mice were crossed with male *loxP* ^+/−^ Cre^+/−^ to generate *loxP* ^+/+^ Cre^+/−^ (*Ager* ^mKO^) mice in the F2 progeny. For breeding, *Ager* ^mKO^ mice were paired with (*loxP* ^+/+^ Cre^−/−^) *Ager* ^*flox*^ mice to avoid Cre^+/+^ homozygosis, which is known to reduce fertility in females. For mouse genotyping, genomic DNA (gDNA) was extracted from ear tips utilizing the PCRBIO Rapid Extract PCR kit according to the manufacturer's instructions. gDNA was subjected to PCR analysis using primers amplifying the *Flox* sequence in the *Ager* gene (498 and 609 bp for WT and floxed, respectively) or *Cre* sequence (248 bp) (protocol 40180 ACTA1‐Cre; Jackson Laboratory). Samples were electrophoresed on 1.3% (*Flox*) or 1.6% (Cre) agarose gel, and the amplification products were revealed with iBright1500 using the non‐mutagenic fluorescence reagent, Novel Juice. To confirm skeletal muscle‐specific deletion of the *Ager* gene, tamoxifen‐treated *Ager* ^*flox*^ and *Ager* ^mKO^ mice were sacrificed, and *tibialis anterior* (TA), *gastrocnemius* (GC), *quadriceps femoris* (QF) and diaphragm (DIA) muscles, heart, lungs, liver and gut were isolated and processed with PureDireX Genomic DNA Isolation kit to extract gDNA. PCR analysis was performed by using a *forward* primer spanning *Ager* exons 1 and 2 and a *reverse* primer spanning *Ager* exons 7 and 8. PCR products (861 and 2105 bp for deleted and not‐deleted *Ager* gene, respectively) were revealed with iBright1500 using Novel Juice reagent.

Eight‐ to 12‐week‐old male *Ager* ^*flox*^, *Ager* ^mKO^ and *Ager* ^−/−^ mice were treated i.p. with tamoxifen (75‐mg/kg bw in corn oil solution) for three consecutive days and injected subcutaneously (s.c.) with LLC cells (1.5 × 10^6^ cells/mouse) or vehicle (control) after 15 days. Mice were monitored daily for survival, body changes, tumour growth and clinical signs until 25 days post‐LLC injection (dpi).

In vivo tumour measurements were taken using a digital calliper, and the tumour volumes were calculated using the formula, Volume (mm^3^) = [long axis (mm) × short axis (mm)^2^]/2. For Kondziela's inverted screen test, each mouse was placed in the centre of a wire mesh screen, the screen was rotated by 180° and the time when the mouse fell off was measured for a maximum of 5 min. Each mouse was evaluated in three trials with 10 min intertrial intervals.

At 25 dpi, animals were sacrificed, and blood was collected. Skeletal muscles, adipose tissue and tumour masses were surgically excised, weighed and collected. TA and GC muscles of one hind limb were divided longitudinally into two equal parts, which were allocated randomly to WB or real‐time PCR analysis. The entire contralateral muscles were processed for histology/immunohistochemistry or proteomic analysis.

Mice were housed under specific pathogen‐free conditions on a 12‐h light/day cycle and raised under a standard mouse diet. Body weights, food intake and clinical signs were monitored daily. Handling was kept to a minimum. Animal procedures followed the 3Rs principles in alignment with the Directive 2010/63/EU of the European Union and approved by the Ethics Committee of the University of Perugia and the Italian Ministry of Health (Authorization #679/2021‐PR).

### 2.3 Western Blotting

Muscle samples were lysed in protein lysis buffer (10‐mM Tris–HCl [pH 7.4], 2.5% v/v sodium dodecyl sulphate [SDS], 100‐mM dithiothreitol [DTT], 200‐mM phenylmethanesulfonyl fluoride [PMSF], 10‐mg/mL aprotinin, 1‐mg/mL pepstatin and 5‐mg/mL leupeptin) and were resolved by SDS‐polyacrylamide gel electrophoresis and transferred to nitrocellulose blots. Blots were blocked with 5% nonfat dried milk and incubated with the primary antibody (O.N. at 4°C) followed by the appropriate HRP‐conjugated secondary antibody (1 h at R.T.). The chemiluminescence of the immunostained bands was visualized using Western Bright Quantum HRP substrate and acquired with iBright 1500. The relative densities of the bands were determined with respect to α‐actinin or total proteins evaluated with No‐stain total Protein Labeling reagent, as indicated.

### 2.4 Real‐Time PCR

Muscle samples were homogenized in TRIsure reagent, and total RNA was extracted following the manufacturer's instructions. Reverse transcription was performed using the PrimeScript RT reagent kit. Real‐time PCR analyses were performed on the QuantStudio 1 Real‐Time PCR system using PowerUp SYBR Green Master Mix. The analyses were performed with QuantStudio Design & Analysis software, in comparison with the TATA box binding protein gene (*Tbp*) as a standard gene.

### 2.5 Histology and Morphometric Evaluation

Formalin‐fixed paraffin‐embedded muscles and tumour masses were cut in 4‐μm sections and processed for haematoxylin/eosin staining. For each muscle or tumour mass, slices at 100‐μm intervals were obtained along the entire length of the sample. Muscles were cut transversely. Slices were analysed with an Olympus BX51 bright‐field microscope equipped with a digital camera. Myofibre cross‐sectional areas (CSAs) and tumour necrosis areas were measured using ImageJ software.

### 2.6 Immunohistochemistry and Immunofluorescence

Formalin‐fixed paraffin‐embedded sections of muscles were deparaffinized and rehydrated in a graded ethanol series. Antigen retrieval was performed by boiling in 10‐mM citric acid buffer (pH 6.0) for 1.5 h. For immunohistochemistry, endogenous peroxidase activity was inhibited by treatment with 3% H_2_O_2_. Sections were blocked with 10% HS in T‐TBS (B.B.) and probed with goat anti‐RAGE primary antibody (1:50 in B.B.) in a humid chamber at 4°C O.N. Sections were incubated with an anti‐goat biotinylated antibody (1:500 in B.B.) for 1 h at R.T. and incubated with ABC kit reagents for 45 min. Incubation with 0.01% DAB and 0.006% H_2_O_2_ in 50‐mM Tris–HCl (pH 7.4) was used to reveal the reaction. Haematoxylin was used to counterstain nuclei, and the sections were dehydrated, mounted with EUKITT and photographed with Olympus BX51 bright‐field microscope equipped with a digital camera. For immunofluorescence, antigen retrieval was performed by treatment with proteinase K (20 μg/mL) before boiling in 10‐mM citric acid buffer (pH 6.0) for 1.5 h. Endogenous peroxidase activity was inhibited by treatment with 1% H_2_O_2_ in methanol for 30 min. Sections were blocked with 3% BSA, 1% glycine in PBS for 1 h and probed with rabbit monoclonal anti‐Myosin Skeletal Slow (EPR22697‐17), anti‐MyHC‐IIa (SC‐71) and anti‐MyHC‐IIb (BF‐F3) antibodies (1:50 in 3% BSA) in a humid chamber at 4°C O.N. Sections were incubated with goat anti‐mouse IgG1 Alexa Fluor 488, goat anti‐mouse IgM (heavy chain) Alexa Fluor 555 or Goat anti‐Rabbit IgG (H + L) Cross‐Adsorbed DyLight 405 antibodies (1:50 in 3% BSA) for 2 h at R.T. DAPI was used to counterstain nuclei. Sections were mounted with ProLong Glass Antifade medium and photographed with Nikon Ti‐E Inverted Fluorescence Motorized Microscope with Spinning Disc. For each section, the entire area was photographed and evaluated for myofibre type distribution.

### 2.7 ELISA for Serum Cytokines

Serum cytokine (IL‐1β, IL‐4, IL‐6, IL‐10, IL‐15, LIF and TNF‐α) levels were measured with a ProcartaPlex MAGPIX Liquid Assay kit according to the manufacturer's instructions and analysed by the specific software.

### 2.8 Proteomic Analysis

GC muscles were lysed with RIPA buffer, disrupted by FastPrep‐24 5G bead‐beating grinder and lysis system. Proteins were precipitated with cold acetone and resuspended. Proteins were reduced in 25 μL of 100‐mM NH_4_HCO_3_ with 2.5 μL of 200‐mM DTT at 60°C for 45 min and alkylated with 10 μL 200‐mM iodoacetamide for 1 h at RT in the dark. Iodoacetamide excess was removed by the addition of 200‐mM DTT, and proteins were digested with trypsin [14]. Digested peptides were dried by Speed Vacuum, desalted and analysed on an Ultimate 3000 RSLC nano coupled directly to an Orbitrap Exploris 480 with a FAIMS pro System. Samples were injected onto a reversed‐phase C18 column (15 cm × 75 μm) and eluted with a gradient of 6% to 95% mobile phase B over 80 min by applying a flow rate of 300 nL/min, followed by an equilibration with 6% mobile phase B for 8 min. Mass spectrometry (MS) scans were performed in the range of m/z 375–1200 at a resolution of 120 000 (at m/z = 200). MS/MS scans were performed by choosing a resolution of 15 000; normalized collision energy of 30%; isolation window of 2 m/z; and dynamic exclusion of 45 s. Two different FAIMS compensation voltages were applied (−45 and −60 V), with a cycle time of 1.5 s per voltage. FAIMS was operated in standard resolution mode with a static carrier gas flow of 4.6 L/min. The acquired raw MS data files were processed and analysed using Proteome Discoverer with Chimerys v3.0.0.757. SequestHT was used as a search engine, and the following parameters were chosen. Database: *Mus musculus* (Uniprot, downloaded on 01‐02‐2023) enzyme: trypsin; max. missed cleavage sites: 2; static modifications: carbamidomethyl (C); dynamic modifications: oxidation (M); precursor mass tolerance: 10 ppm; fragment mass tolerance: 0.02 Da. Only peptides and proteins with FDR value < 0.01 were reported. The abundance of identified peptides was determined by label‐free quantification (LFQ) using a match between runs. Statistical analyses and *t*‐tests were performed on protein abundances using MetaboAnalyst software [15]. The modulated proteins were analysed through ShinyGO 0.80 enrichment tool. The significant overlapping changes were determined by Venn diagram (Bioinformatics & Evolutionary Genomics). Heatmaps and volcano plots were obtained with SRplot online tool.

### 2.9 Clinical Samples

Patients with a new diagnosis of pancreatic cancer, eligible for surgical tumour resection, and controls undergoing surgery for non‐malignant diseases (inguinal hernia), were consecutively enrolled at the Department of Translational and Precision Medicine, Sapienza University of Rome, Italy. The inclusion criteria of the study were age ≥ 18 years; recent diagnosis of cancer (≤ 4 weeks); not having received anticancer or anti‐inflammatory treatments before surgery; and capability to give informed consent. Patients with coexisting conditions inducing malnutrition, such as chronic kidney diseases, infections, liver failure, heart failure, rheumatologic disorders, clear signs of malabsorption or intestinal occlusion and dysphagia, were excluded. The study was conducted according to the Declaration of Helsinki and approved by the local Ethics Committee. Written informed consent was obtained by all the participants enrolled in the study. At the first visit, we collected demographic information, patients' medical history and data on the tumour stage and histology. We registered body weight (kg) and height (m), calculated the body mass index (BMI, kg/m^2^) and asked for usual weight and involuntary body weight loss in the previous 6 months. Following standardized criteria, pre‐cachexia was defined as ≤ 5% weight loss over the past 6 months, with anorexia (FAACT score ≤ 30) and inflammation (CRP > 10 mg/L) [16]. Presence of CC was diagnosed according to Fearon's criteria [17], as > 5% weight loss over the past 6 months, or BMI < 20 kg/m^2^ and > 2% weight loss. Rectus abdominis muscle biopsies were obtained during the first phase of the surgical procedure in both cancer patients and controls (approximately 1 cm^3^). Specimens were immediately frozen in liquid nitrogen and stored at −80°C.

### 2.10 Reagents and Resources

See Table S1.

### 2.11 Statistical Analysis

The number of animals used is specified in each experiment. Counts were performed by three independent operators blind to treatments. Western blotting, ELISA and real‐time PCR analyses were performed at least in triplicate for each biological sample. Representative experiments and images are shown unless stated otherwise. Data are presented as mean ± SEM for each experimental group. Statistical significance was assessed using Student's *t*‐test and one‐ and two‐way analysis of variance (ANOVA). A *p*‐value < 0.05 was considered statistically significant. Statistical analyses were performed using GraphPad Prism 10 software.

### 2.12 Data and Code Availability

The MS proteomics data have been deposited in the ProteomeXchange Consortium via the PRIDE partner repository with the dataset identifier PXD060768 (https://www.ebi.ac.uk/pride/archive/projects/PXD060768).

Supplemental tables and figures are available as Supporting Information.

Any additional information, including unprocessed data required to reanalyse the data reported in this paper, is available from the lead contact (guglielmo.sorci@unipg.it) upon request.

## 3 Results

### 3.1 Generation of a Muscle‐Specific Conditional RAGE Knock‐Out (*Ager* ^mKO^) Mouse Model

**FIGURE 1:**
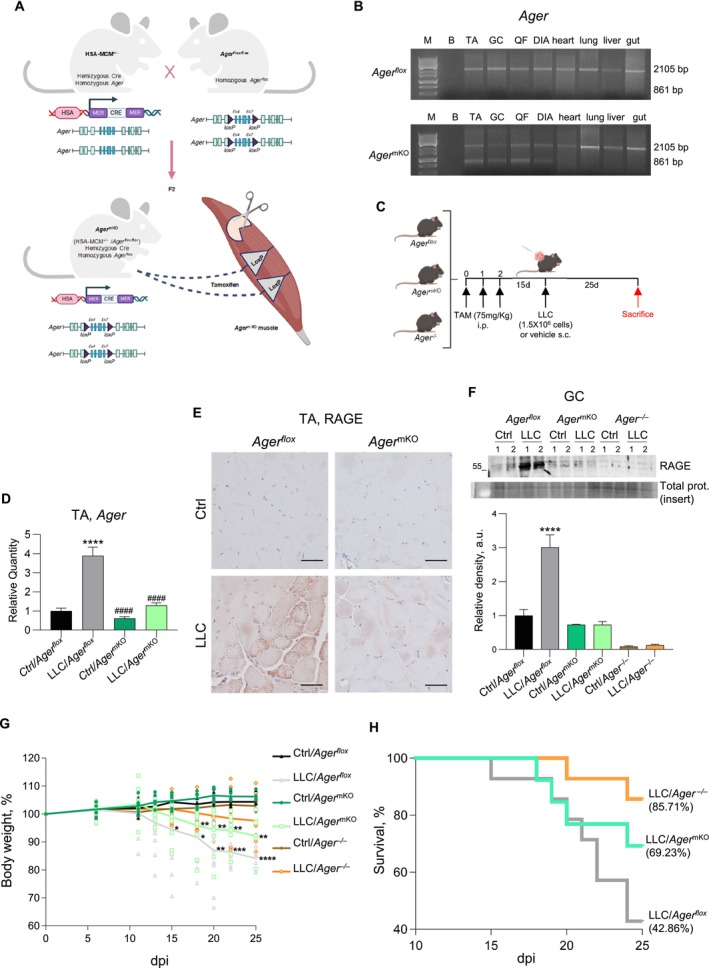
*Ager* ^mKO^ mice were more resistant to CC and survived longer than control mice. (A) Schematic representation of the generation of *Ager* ^mKO^ mice by crossing HSA‐MCM mice (with tamoxifen‐inducible Cre recombinase under the control of myofibre‐specific HSA promoter) with *Ager* ^*flox*^ mice (loxP flanked *Ager* genomic region, exons 4 to 7). (B) PCR analysis of several organs and muscles of *Ager* ^mKO^ and *Ager* ^*flox*^ mice after injection with tamoxifen. The 861‐bp PCR products indicate deletion of the *Ager* exons 4 to 7. TA, *tibialis anterior*; GC, gastrocnemius; QF, *quadriceps femoris*; DIA, diaphragm. M, marker; B, blank. (C) Schematic representation of the cachexia LLC model with *Ager* ^*flox*^, *Ager* ^mKO^ and *Ager* ^−/−^ mice. Control mice were injected s.c. with vehicle. (D–F) RAGE expression in TA or GC muscles of LLC/*Ager* ^*flox*^, LLC*/Ager* ^mKO^ and LLC/*Ager* ^−/−^ mice (*n* = 8) at 25 dpi was analysed by real‐time PCR (D), immunohistochemistry (E) or Western blotting (F) in comparison with their internal controls (Ctrl; *n* = 6). *Tbp* was used as a housekeeping gene (D). Reported are the average relative densities of RAGE bands with respect to total proteins and Ctrl/*Ager* ^*flox*^ mice (F) (see Figure S2A for total protein staining). Statistical significance with respect to internal controls is reported (F). Shown are representative images (B,E,F). Bars (E), 50 μm. (G,H) Body weight changes (G) and Kaplan–Meier curve (H) of LLC/*Ager* ^*flox*^ (*n* = 14), LLC/*Ager* ^mKO^ (*n* = 13) and LLC/*Ager* ^−/−^ (*n* = 14) mice. The body weight changes of control animals (Ctrl; *n* = 6 each group) are reported for comparison (G). The survival rates at 25 dpi are indicated (H). Data are mean ± SEM. One‐way ANOVA (D,F) or two‐way ANOVA (G); *p < 0.05, **p < 0.01, ***p < 0.001, ****p < 0.0001 vs. internal Ctrl (if not otherwise indicated); ^####^ *p* < 0.001 vs. LLC/*Ager* ^*flox*^.

To understand the role of RAGE re‐expressed at the muscle level in CC conditions, we generated a conditional *Ager* ^mKO^ mouse model in which RAGE expression is selectively ablated in adult skeletal myofibres. To this end, a Cre‐*loxP* system was used in which *Ager* ^*flox*^ mice [12] were crossed with tamoxifen‐inducible HSA‐MCM mice expressing MerCreMer double fusion protein under the control of human alpha‐skeletal actin (HSA or *ACTA1*), which is specifically expressed in both slow‐ and fast‐twitch adult myofibres [13] (Figure 1A). The specific deletion of exons 4 to 7 of the *Ager* gene in skeletal muscles of *Ager* ^mKO^ mice was confirmed by genomic PCR analysis of several tissues in comparison with control, *Ager* ^*flox*^ mice after treatment with tamoxifen (Figure 1B). The persistence of the PCR product (2105 bp) related to wild‐type RAGE in muscles of *Ager* ^mKO^ mice has to be attributed to cells other than myofibres present in muscle tissue (e.g., endothelial cells, fibroadipogenic progenitors and pericytes), on which the Cre‐*loxP* system used is ineffective. *Ager* ^mKO^ mice are viable and fertile and do not display an overt phenotype in basal conditions, as expected, considering the normal phenotype reported in the complete absence of RAGE (*Ager* ^−/−^ mice) [11]. No differences were found between tamoxifen‐injected 3‐ or 6‐month‐old *Ager* ^mKO^ and age‐matched *Ager* ^*flox*^ mice in terms of body and muscle weights (Figure S1A–C), and muscle performance in the absence or presence of tamoxifen treatment (Figure S1D).

### 3.2 *Ager* ^mKO^ Mice Undergo Reduced Loss of Body Weight and Survive Longer Than Control Mice in Cancer Conditions

Subcutaneous (s.c.) injection of LLC cells in mice is a widely accepted experimental model of CC [18]. We injected LLC cells s.c. in *Ager* ^*flox*^ (LLC/*Ager* ^*flox*^), *Ager* ^mKO^ (LLC/*Ager* ^mKO^) and *Ager* ^−/−^ (LLC/*Ager* ^−/−^) mice previously treated with tamoxifen, and sacrificed the animals after 25 days (Figure 1C). At 25 dpi, TA muscles of LLC/*Ager* ^*flox*^ mice showed increased levels of *Ager* mRNA and the appearance of RAGE‐positive myofibres compared with controls (Figure 1D,E), in line with the results obtained in WT mice [10]. No RAGE re‐expression was observed in muscles of LLC/*Ager* ^mKO^ mice at the same time point by real‐time PCR analysis and immunohistochemistry (Figure 1D,E). WB analysis performed on muscles of the three animal models showed similar amounts of RAGE in *Ager* ^*flox*^ and *Ager* ^mKO^ mice in basal conditions, and further confirmed the inability to re‐express RAGE by muscles of tamoxifen‐treated *Ager* ^mKO^ mice in the presence of cancer (Figures 1F and S2A).

LLC/*Ager* ^mKO^ mice lost body weight over time to a lesser extent than LLC/*Ager* ^*flox*^ mice (culminating in 7.5% ± 2.5% and 15.1% ± 1.3% reduction in body weights, respectively, at 25 dpi), and started losing weight at 18 dpi, in contrast with LLC/*Ager* ^*flox*^ mice, which started losing weight at 15 dpi (Figure 1G). Noteworthy, LLC/*Ager* ^mKO^ mice survived longer than LLC/*Ager* ^*flox*^ mice, with 69.2% and 42.9% mice alive, respectively, at 25 dpi (Figure 1H). However, the highest protection against CC was observed in the total absence of RAGE since LLC/*Ager* ^*−/−*^ mice showed no significant loss of body weight, and an 85.7% survival rate at 25 dpi (Figure 1G,H), in line with published results [10].

No significant differences were found in terms of weights, histology and extent of necrotic areas in LLC masses isolated from the three animal groups after sacrifice (Figure S3), suggesting that the restrained CC observed in LLC/*Ager* ^mKO^ mice was mainly linked to the absence of RAGE at the muscle level.

### 3.3 RAGE Ablation at Muscle Level Restrains Cancer‐Induced Muscle Wasting

**FIGURE 2:**
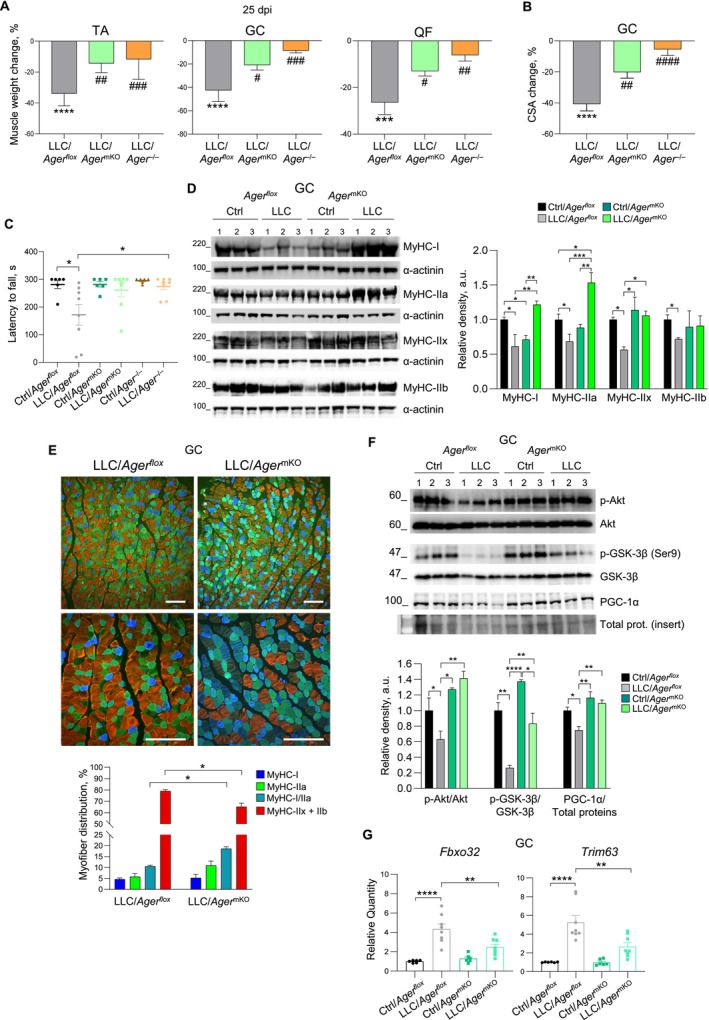
Deletion of RAGE in myofibres prevents cancer‐induced muscle wasting. (A–G) Tamoxifen‐treated *Ager* ^*flox*^, *Ager* ^mKO^ and *Ager* ^−/−^ mice were injected s.c. with LLC cells (*n* = 8) or vehicle as a control (*n* = 6), and sacrificed at 25 dpi. (A) Weight changes of muscles. TA, *tibialis anterior*; GC, gastrocnemius; QF, *quadriceps femoris*. (B) Changes in the average myofibre cross‐sectional areas (CSAs) of GC muscles of LLC/*Ager* ^*flox*^, LLC/*Ager* ^mKO^ and LLC/*Ager* ^−/−^ mice at 25 dpi compared with their non‐tumour‐bearing internal controls. (C) Muscle functionality evaluated by Kondziela's inverted screen test. Each point represents an individual mouse. (D) Representative Western blot images of slow MyHC‐I and fast MyHC‐IIa, ‐IIx and ‐IIb in GC muscles of *Ager* ^*flox*^ and Ager^mKO^ mice in the absence (Ctrl) or presence (LLC) of tumour at 25 dpi (*left panel*). Reported are the relative densities with respect to α‐actinin (*right panel*). (E) Representative immunofluorescence images of GC muscles of LLC/*Ager* ^*flox*^ and LLC/*Ager* ^mKO^ mice at 25 dpi stained with specific antibodies for MyHC‐I (*blue*), MyHC‐IIa (*green*) and MyHC‐IIb (*red*) (*upper panel*). *Cyan* colour indicates hybrid MyHC‐I/IIa myofibres. Reported are the percentages of single‐positive or MyHC‐I/IIa double‐positive myofibres (*lower panel*). Bars, 200 μm. (F) Representative Western blot images of total and phosphorylated Akt and GSK‐3β, and PGC‐1α in GC muscles of *Ager* ^*flox*^ and *Ager* ^mKO^ mice injected or not with LLC cells at 25 dpi (*upper panel*). Reported are the relative densities with respect to the total form of kinases or total proteins (*lower panel*) (see Figure S2B for total protein staining). (G) Real‐time PCR for *Fbxo32* and *Trim63* in GC muscles of *Ager* ^*flox*^ and *Ager* ^mKO^ mice injected or not with LLC cells at 25 dpi. *Tbp* was used as a housekeeping gene. Data are mean ± SEM. One‐way ANOVA; *p < 0.05, **p < 0.01, ***p < 0.001, ****p < 0.0001 vs. internal Ctrl (if not otherwise indicated); ^#^ *p* < 0.05, ^##^ *p* < 0.01, ^###^ *p* < 0.001, ^####^ *p* < 0.0001 vs. LLC/*Ager* ^*flox*^.

Since decreased skeletal muscle mass is a clinical sign of CC [1, 17], we weighed muscles isolated from the three different animal models at the end of the experimentation (i.e., 25 dpi). While hind‐limb (TA, GC and QF) muscles of LLC/*Ager* ^*flox*^ mice were markedly reduced in weight compared with Ctrl/*Ager* ^*flox*^ mice, muscles isolated from LLC/*Ager* ^mKO^ and LLC/*Ager* ^*−/−*^ mice showed only slight, not significant, mass reductions compared with their internal controls (Figure 2A). Morphologically, muscles of LLC/*Ager* ^*flox*^ mice showed an ~40% average reduction in myofibre CSA compared with internal controls, whereas the myofibre CSAs of *Ager* ^mKO^ mice were not significantly affected by the presence of the tumour, similarly to *Ager* ^*−/−*^ mice (Figures 2B and S4A–C). Accordingly, LLC/*Ager* ^mKO^ and LLC/*Ager* ^*−/−*^ mice, but not LLC/*Ager* ^*flox*^ mice, preserved their muscle strength at 25 dpi, as evaluated by Kondziela's inverted screen test (Figure 2C).

LLC/*Ager* ^*flox*^ and LLC/*Ager* ^mKO^ mice lost adipose tissue (inguinal [iWAT] and epididymal [eWAT] white adipose tissues) to a similar extent, in comparison with their internal controls (Figure S5), suggesting that the reduced loss in body weight observed in LLC/*Ager* ^mKO^ mice (Figure 1G) was mainly related to the preservation of muscle mass.

### 3.4 Akt‐GSK‐3β‐PGC‐1α Signaling and Increased MyHC‐I and ‐IIa Characterize Muscles Lacking RAGE in Cancer Conditions

WB analysis performed with the use of specific antibodies to the different MyHC isoforms (i.e., slow‐twitch oxidative MyHC‐I, fast‐twitch oxidative/glycolytic MyHC‐IIa and fast‐twitch glycolytic MyHC‐IIx and ‐IIb) showed that GC muscles of LLC/*Ager* ^*flox*^ mice were characterized by reduced amounts of both slow and fast MyHC isoforms compared with control mice at 25 dpi (Figure 2D). On the contrary, LLC/*Ager* ^mKO^ muscles showed dramatically increased levels of MyHC‐I and ‐IIa (71.8% and 73.9% increase, respectively), which were accompanied by preserved amounts of MyHC‐IIx and ‐IIb, in comparison with muscles of Ctrl/*Ager* ^mKO^ mice (Figure 2D). In line with these observations, immunofluorescence staining revealed a 76.3% increase in hybrid double‐positive MyHC‐I/IIa myofibres in muscles of LLC/*Ager* ^mKO^ compared with LLC/*Ager* ^*flox*^ mice, accompanied by a concomitant reduction in IIx and IIb myofibres (Figure 2E). Altogether, these results pointed to a myofibre remodeling induced by the absence of RAGE at myofibre level in cancer conditions.

Looking for a mechanistic explanation, we found that the activation (phosphorylation) state of the anabolic kinase, Akt (p‐Akt), was strongly reduced in GC muscles of *Ager* ^*flox*^ mice in the presence of cancer, whereas physiological levels of p‐Akt were maintained in muscles of tumour‐bearing *Ager* ^mKO^ mice (Figure 2F). In LLC/*Ager* ^mKO^ mice, we also found higher levels of phosphorylated glycogen synthase kinase (GSK)‐3β, a kinase whose phosphorylation on Ser9 by p‐Akt results in its inhibition and reduced degradation of peroxisome proliferator‐activated receptor gamma coactivator (PGC)‐1α, which has a role in protecting skeletal muscle from atrophy [19]. Indeed, the higher p‐GSK‐3β/GSK‐3β ratio was accompanied by higher amounts of PGC‐1α in the muscles of LLC/*Ager* ^mKO^ compared with LLC/*Ager* ^*flox*^ mice (Figure 2F). In accordance, *Fbxo32* and *Trim63* atrogenes appeared strongly induced in GC muscles of LLC/*Ager* ^*flox*^ mice, but not significantly increased in LLC/*Ager* ^mKO^ muscles at 25 dpi (Figure 2G). Similar results were obtained in TA muscles isolated from the different animal groups in terms of MyHC‐II, p‐Akt and p‐GSK‐3β expressions, and atrogenes levels (Figure S4D,E). Thus, the absence of RAGE at muscle level translates into the maintenance of a physiological activation state of the Akt‐GSK‐3β‐PGC‐1α pathway in cancer conditions, which might contribute to protecting muscles against cancer‐induced wasting.

### 3.5 RAGE Ablation at Muscle Level Translates Into Reduced Systemic and Muscle Inflammation

**FIGURE 3:**
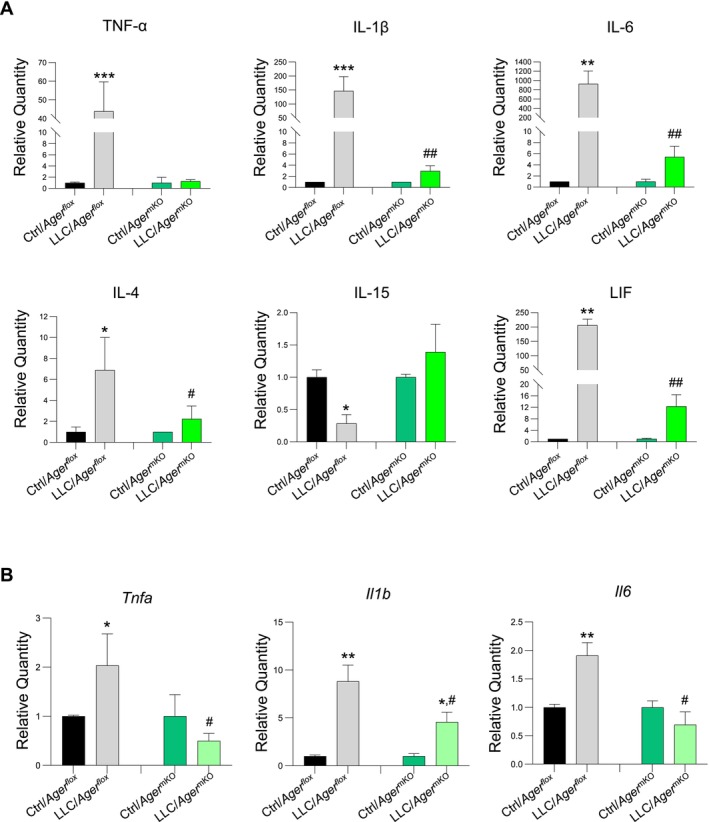
*Ager* ^mKO^ mice show reduced muscle and serum inflammation in cancer conditions. (A,B) Serum level changes of interleukins, TNF‐α and LIF (A), and expression levels of *Tnfa*, *Il1b* and *Il6* in GC muscles (B) of *Ager* ^*flox*^ and *Ager* ^mKO^ mice injected or not with LLC cells (*n* = 6 each group) at 25 dpi as evaluated by ProcartaPlex array or real‐time PCR, respectively. *Tbp* was used as a housekeeping gene (B). Data are mean ± SEM. One‐way ANOVA; *p < 0.05, **p < 0.01, ***p < 0.001 vs. internal Ctrl; ^#^ *p* < 0.05, ^##^ *p* < 0.01 vs. LLC/*Ager* ^*flox*^.

**FIGURE 4:**
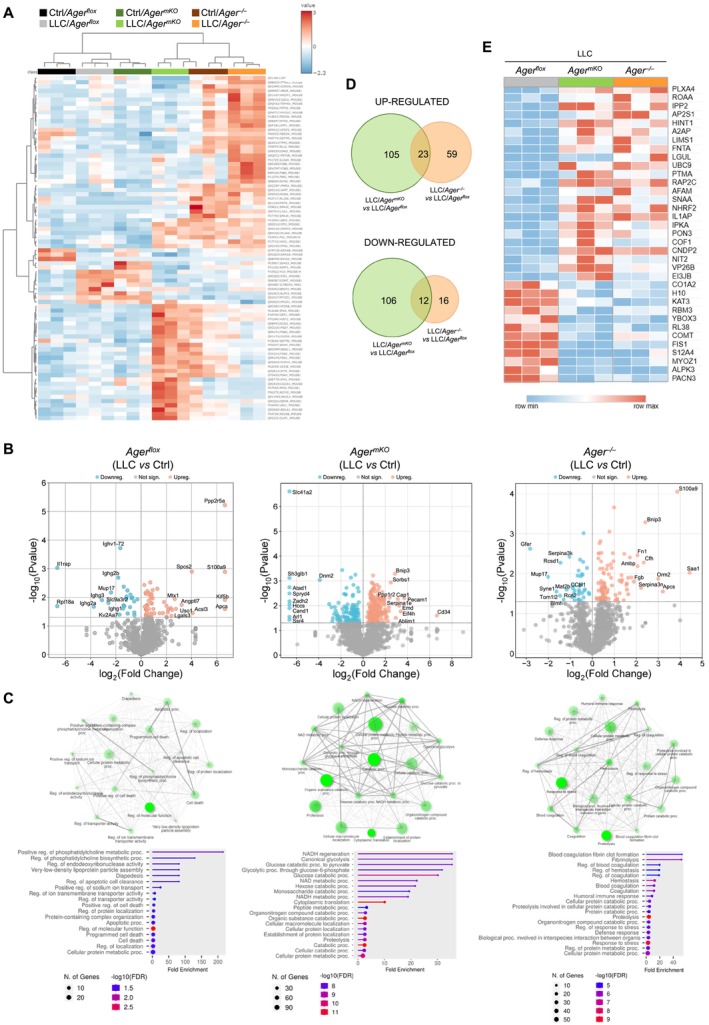
Muscles of *Ager* ^*flox*^, *Ager* ^mKO^ and *Ager* ^−/−^ mice reveal distinct proteomic signatures in cancer conditions. (A) Heatmap of the modulated proteins in GC muscles of *Ager* ^flox^, *Ager* ^mKO^ and *Ager* ^−/−^ mice injected or not with LLC cells (*n* = 3 each group) at 25 dpi. (B) Volcano plots showing the significantly (*p* < 0.05) downregulated and upregulated proteins in LLC‐bearing mice compared with internal controls. The 10 most downregulated and upregulated proteins are indicated. (C) ShinyGO analysis of proteins modulated in muscles (LLC vs. internal control) using the gene ontology biological process database. Networks (*upper panels*) and pathways (*lower panels*) are reported. (D) Venn diagrams of the proteins modulated (*p* < 0.05) in muscles of LLC*/Ager* ^*mKO*^ or LLC/*Ager* ^*−/−*^ vs. LLC/*Ager* ^*flox*^ mice (D). (E) Heatmap of proteins modulated in common in GC muscles of LLC*/Ager* ^*mKO*^ and LLC/*Ager* ^*−/−*^ vs. LLC/*Ager* ^*flox*^ mice.

The inflammatory response has a determinant role in the onset and progression of CC [1, 3], and *Ager* ^*−/−*^ mice are characterized by restrained systemic inflammation and cachexia in cancer conditions [10]. Thus, we investigated the effects of RAGE ablation at myofibre level on the serum cytokine profile in tumour‐bearing mice at 25 dpi. We found dramatically lower levels of the pro‐cachectic factors, tumour necrosis factor (TNF)‐α, interleukin (IL)‐1β, IL‐6 and leukaemia inhibitory factor (LIF) in LLC/*Ager* ^mKO^ mice compared with LLC/*Ager* ^*flox*^ mice. LLC/*Ager* ^mKO^ mice maintained physiological serum levels of IL‐15, a myokine that protects against muscle protein catabolism [20], and that was found to be reduced in the serum of LLC‐bearing *Ager* ^*flox*^ mice (Figure 3A). Lower serum levels of the anti‐inflammatory cytokine, IL‐4, were detected in LLC/*Ager* ^mKO^ in comparison with LLC/*Ager* ^*flox*^ mice (Figure 4A), likely as a mirror and consequence of a reduced inflammatory state. The restrained systemic inflammation observed in LLC/*Ager* ^mKO^ mice was accompanied by reduced gene expression of the pro‐cachectic factors, TNF‐α, IL‐1β and IL‐6 in their muscles (Figure 3B). These results supported a main role of RAGE expressed at myofibre level in sustaining both muscle and systemic inflammation.

### 3.6 Muscles of Tumour‐Bearing *Ager* ^*flox*^, *Ager* ^mKO^ and *Ager* ^*−/−*^ Mice Show Distinct Proteomic Signatures

A pan‐investigation of the proteomic profiles of GC muscles of *Ager* ^*flox*^, *Ager* ^mKO^ and *Ager* ^−/−^ mice injected or not with LLC cells at 25 dpi was performed using quantitative MS. More than 1500 unique proteins were quantified. The hierarchical clustering heatmap clearly showed distinct proteomic profiles associated with the different animal groups (Figure 4A). The monovariate statistical analysis identified 59, 197 and 88 proteins upregulated, and 25, 118 and 37 proteins downregulated in LLC/*Ager* ^*flox*^, LLC/*Ager* ^mKO^ and LLC/*Ager* ^−/−^ mice, respectively, in comparison with their internal controls (Figure 4B).

ShinyGO analysis of the gene ontology biological processes showed ‘Cellular protein metabolic processes’ as the only term shared by LLC/*Ager* ^*flox*^, LLC/*Ager* ^−/−^ and LLC/*Ager* ^mKO^ mice vs. internal controls (Figure 4C; Table S2), suggesting muscle metabolism alteration as a unifying process in cancer conditions. Among the proteins belonging to that term, HMGB1, which is a RAGE ligand associated with inflammation and a recognized cachexigenic factor [10], emerged as upregulated in muscles of LLC/*Ager* ^*flox*^ but not LLC/*Ager* ^mKO^ and LLC/*Ager* ^−/−^ mice. Terms related to cell death and apoptosis were the most represented in LLC/*Ager* ^*flox*^ mice. They included proteins known to promote apoptosis in muscle or other tissues, such as S100A9, HMGB1, TOMM40 (mitochondrial import receptor subunit TOM40 homologue), ANP32E (acidic leucine‐rich nuclear phosphoprotein 32 family member E), PTPA (serine/threonine‐protein phosphatase 2A activator), CTSD (cathepsin D) and CAPN2 (calpain‐2 catalytic subunit) (Figure 4C; Table S2). No terms related to cell death and apoptosis emerged in muscles of LLC/*Ager* ^mKO^ or LLC/*Ager* ^−/−^ mice.

An overall metabolic alteration characterized the proteomic profile of LLC/*Ager* ^mKO^ muscles, which showed increased amounts of several enzymes involved in glycolysis and glucose catabolism. They included ALDOA (fructose‐bisphosphate aldolase A), ENO1B (enolase 1), ENO3 (enolase 3), HK1 (hexokinase‐1), LDHA (lactate dehydrogenase A), PGK1 (phosphoglycerate kinase 1) and PKM (pyruvate kinase) (Figure 4C; Table S2), thus covering the entire glycolytic cascade [21]. Since no proteins or terms related to mitochondrial dysregulation emerged from the proteomic profile of LLC/*Ager* ^mKO^ muscles, this scenario was reminiscent of a metabolic response known as aerobic glycolysis (or Warburg effect) through which cells utilize glycolysis to rapidly regenerate ATP even in the presence of sufficient oxygen and active mitochondria [22]. The increased expression of several of those enzymes involved in the Warburg effect (i.e., ALDOA, LDHA, PGK1 and PKM1/2) was confirmed in LLC/*Ager* ^mKO^ muscles, in comparison with muscles of Ctrl/*Ager* ^mKO^ mice, by WB analysis (Figure S6).

Finally, terms related to fibrinolysis, blood coagulation, response to stress and the immune response, along with metabolic alteration, characterized muscles of LLC/*Ager* ^*−/−*^ mice (Figure 4C; Table S2).

To have additional information about the mechanisms contributing to confer resistance to CC, we compared the proteomic profiles of GC muscles of LLC/*Ager* ^−/−^ or LLC/*Ager* ^mKO^ mice with those of LLC/*Ager* ^*flox*^ mice and found 23 proteins upregulated and 12 proteins downregulated in common in mice with total or muscle ablation of RAGE (Figure 4D,E). Among the proteins upregulated in common, several antioxidant enzymes appeared, including LGUL (lactoylglutathione lyase/glyoxalase 1), PON3 (serum paraoxonase/lactonase 3) and CNDP2 (cytosolic non‐specific dipeptidase/carnosine dipeptidase II), and the SUMO‐conjugating enzyme UBC9, whose expression is typically associated with slow‐twitch muscles [23]. On the contrary, FIS1 (mitochondrial fission 1 protein), which is involved in mitochondrial fission and cell death [24], and MYOZ1 (myozenin 1/calsarcin‐2), which is mainly expressed in fast‐twitch muscle fibres [25], were found downregulated in both LLC/*Ager* ^−/−^ and LLC/*Ager* ^mKO^ compared with LLC/*Ager* ^*flox*^ mice (Figure 4E).

### 3.7 Muscles of Pre‐Cachectic and Cachectic Patients Express Increased Amounts of RAGE and Show Reduced PGC‐1α Signaling

**FIGURE 5:**
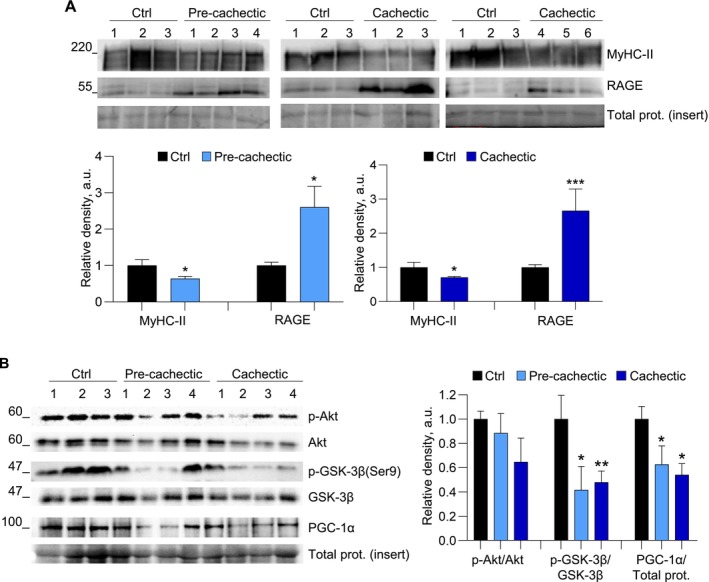
Muscles of pre‐cachectic and cachectic cancer patients express increased amounts of RAGE and reduced PGC‐1α signaling. (A,B) Sample biopsies of *rectus abdominis* muscles derived from clinically diagnosed pre‐cachectic (*n* = 4) or cachectic (*n* = 6) cancer patients, and control subjects (Ctrl; *n* = 3) were analysed for MyHC‐II and RAGE expression (A; *upper panels*), or total and phosphorylated Akt and GSK‐3β, and PGC‐1α expression (B; *upper panels*). Reported are the average relative densities with respect to total Akt, total GSK‐3β or total proteins (A,B; *lower panels*; see Figure S7 for total protein stainings). Student's *t*‐test (A) or one‐way ANOVA (B); *p < 0.05, **p < 0.01, ***p < 0.001 vs. Ctrl.

*Rectus abdominis* muscle biopsies derived from clinically diagnosed pancreatic carcinoma pre‐cachectic (*n* = 4) or cachectic (*n* = 6) patients and control subjects (*n* = 3) were analysed for MyHC‐II and RAGE expression by WB. Muscles of cancer patients in either the pre‐cachectic or cachectic stage were characterized by reduced amounts of MyHC‐II and a dramatically increased expression of RAGE (Figures 5A and S7A), suggesting that the re‐expression of RAGE in muscles is an early event in CC, and that a condition of RAGE hyperstimulation also establishes in muscles of cancer patients undergoing cachexia. Interestingly, muscles of these patients showed a reduced activation of the Akt‐GSK‐3β‐PGC‐1α pathway, since we found reduced p‐Akt (although not statistically significant) and p‐GSK‐3β and reduced amounts of PGC‐1α, especially in the cachectic stage, in comparison with healthy controls (Figures 5B and S7B). These results are in line with those obtained in LLC‐bearing mice and suggest the link between muscular RAGE activity and the reduced expression of PGC‐1α as a common mechanism in sustaining muscle wasting in cancer conditions.

## 4 Discussion

The loss of body and skeletal muscle weight is the main hallmark of CC, an unsolved multifactorial syndrome affecting about half of patients with late‐stage cancer. In particular, the loss of skeletal muscle tissue impairs cancer patients' quality of life and limits the efficacy of anticancer therapies [1, 2]. Excessive protein degradation and decreased protein synthesis in response to systemic inflammation and circulating CCFs are considered the main causes of muscle loss in CC [3], and emerging evidence indicates that the muscle environment is likely to contribute significantly to the cachexia syndrome [4].

We focused on RAGE as an intriguing candidate receptor for potential CC therapeutic approaches since RAGE, besides being expressed in the immune cells participating in the inflammatory response, is re‐expressed in atrophying myofibres in cancer conditions. Moreover, high serum levels of RAGE ligands (e.g., S100B and HMGB1) together with pro‐inflammatory cytokines characterize CC, potentially leading to a hyperstimulation of RAGE, thus fostering the inflammatory state. Indeed, total genetic ablation of RAGE translates into reduced CCF serum levels, delayed loss of muscle mass and strength and increased survival in tumour‐bearing mice [10]. However, to design an efficacious RAGE‐centred therapeutic approach against cancer‐induced muscle wasting, it is important to understand the specific contribution to the onset and progression of CC of the RAGE expressed in the different host compartments, with particular regard for muscle tissue.

Here, we show that the absence of RAGE only at myofibre level translates into a restrained cachectic phenotype and prolonged survival in LLC‐bearing animals, with no significant effects on tumour growth. *Ager* ^mKO^ mice lost lower body weight compared to *Ager* ^*flox*^ mice and maintained their muscle masses in the presence of cancer until 25 dpi, suggesting that RAGE activation in myofibres plays a crucial role in inducing cancer‐dependent muscle wasting. However, the total ablation of RAGE (LLC/*Ager* ^−/−^ mice) resulted in the highest survival rate and body weight preservation, indicating that a host non‐muscle (likely immune) component has a role in promoting CC besides skeletal muscle tissue.

The large majority of studies reported unchanged distribution between type I and type II myofibres in skeletal muscles of both animal models of CC and cachectic cancer patients in comparison with their non‐cachectic cancer or healthy counterparts [26]. Nevertheless, an increase in the percentages of type IIa and/or IIb myofibres has been reported in muscles of CC models, mainly in the soleus [26], suggesting that myofibre type transition is dependent on the muscle's physiological function and extent of cachexia. We found that, contrary to muscles of *Ager* ^*flox*^ mice, muscles of *Ager* ^mKO^ mice showed an increased expression of MyHC‐I and ‐IIa and increased percentages of hybrid MyHC‐I/IIa myofibres in the presence of cancer. This is likely to contribute to the maintenance of muscle mass in these animals since it has been reported that slow‐twitch myofibres are more resistant to CC, and analysis of muscle biopsies from cachectic experimental models and cancer patients showed a prevalence of atrophy in the fast type II myofibres [27]. This is the reason why the soleus muscle, which is mainly made of type I myofibres, is more resistant to cancer‐induced muscle wasting. Notably, the preservation of MyHC‐IIx and ‐IIb and the increased amounts of MyHC‐I and ‐IIa found in muscles of LLC/*Ager* ^mKO^ mice indicate that a selective protein synthesis is activated in these muscles in a condition in which catabolic processes are ongoing, as suggested by the proteomic analysis.

The protection from atrophy observed in slow‐twitch muscles has been linked to their high levels of PGC‐1α, which characterizes myofibres with high mitochondrial oxidative metabolism. PGC‐1α protects muscles from protein degradation hampering the binding of FoxO3, a transcription factor that activates both UPS and ALS, to the atrogin1 promoter, thus inhibiting atrogin1 transcription. A reduction in PGC‐1α expression has been reported in different atrophying conditions, such as denervation, diabetes, renal failure and CC [19]. Here, we show that, contrary to muscles of LLC/*Ager* ^*flox*^ mice, in which a significant downregulation of PGC‐1α together with strong upregulation of atrogin‐1 and MuRF1 is induced by the tumour presence, muscles of LLC/*Ager* ^mKO^ mice do not downregulate PGC‐1α levels and do not upregulate atrogenes expression. LLC/*Ager* ^mKO^ muscles also maintained basal phosphorylation levels of the anabolic kinase, Akt.

Looking for a potential link between Akt and PGC‐1α, we considered that (i) Akt is involved in the phosphorylation (on Ser9) and subsequent inhibition of GSK‐3β [28], whose activity is required for the induction of muscle atrophy and whose inhibition prevents dexamethasone‐induced atrogenes upregulation in myotubes [29]; (ii) PGC‐1α is degraded by the proteasome after phosphorylation and ubiquitination by GSK‐3β activated by oxidative stress [30]; and (iii) the inhibition of GSK‐3β protects against the decrease of PGC‐1α levels induced by hindlimb unloading [31]. Our results suggest that an Akt/GSK‐3β/PGC‐1α pathway is still active in muscles lacking RAGE in cancer conditions, in which Akt activity results in the inhibition of GSK‐3β leading to the maintenance of physiological levels of PGC‐1α, thus contributing to the resistance to CC. On the converse, RAGE signaling is likely to sustain cancer‐induced muscle atrophy by restraining Akt activity, leading to the activation of GSK‐3β, which inhibits protein synthesis and promotes protein degradation [31]. Interestingly, ~30% reduction in the GSK‐3β phosphorylation extent at Ser9 has been reported in muscles of cachectic patients [32].

Since PGC‐1α is a major regulator of the phenotypic adaptation to physical exercise, consisting of a fast‐to‐slow myofibre switch [19], and LLC‐bearing mice undergoing treadmill exercise partially recover muscle mass and strength by increasing PGC‐1α levels [33], the inhibition of RAGE could also be considered for exercise‐mimetic approaches in cachectic patients with compromised mobility.

The proteomic analysis suggested additional factors linked to the absence of RAGE at muscle levels, which might concur to restraining muscle wasting in cancer conditions by promoting myofibre remodeling. In comparison with muscles of LLC/*Ager* ^*flox*^ mice, LLC/*Ager* ^mKO^ and LLC/*Ager* ^−/−^ muscles were characterized by (i) higher amounts of the SUMO‐conjugating enzyme UBC9, which is highly expressed in the atrophy‐resistant slow‐twitch muscles and involved in the determination of myofibre type specificity [23], and (ii) reduced amounts of MYOZ1 (myozenin‐1/calsarcin‐2), which is expressed exclusively by fast‐twitch muscles where it inhibits the calcineurin/NFAT pathway responsible for fast‐to‐slow myofibre transition [25, 34]. Altogether, these results suggest a role of RAGE in determining myofibre specificity, which is supported by the observation that muscles of *Ager* ^−/−^ mice are characterized by increased amounts of MyHC‐I in basal conditions [13].

The same proteomic comparison showed upregulation of several antioxidant enzymes (i.e., LGUL, PON3 and CNDP2) and downregulation of FIS1 in muscles of LLC/*Ager* ^−/−^ and LLC/*Ager* ^mKO^ mice compared with LLC/*Ager* ^*flox*^ mice. Increased levels of reactive oxygen species (ROS) and oxidation‐dependent protein modifications are typical hallmarks of cachectic patients' muscles [35], and oxidative stress results in enhanced mitochondrial fission. An increased expression of FIS1, together with altered mitochondrial morphology, has been observed in muscles of cachectic animals and patients [35, 36]. Thus, our results indicate that an environment characterized by reduced oxidative stress establishes in muscles lacking RAGE in cancer conditions. Moreover, the reduced expression of FIS1 is likely to contribute to the protection of muscles from wasting, since the genetic silencing of FIS1 in skeletal muscles prevented the mass loss induced by atrophying amounts of FoxO3 [35, 36].

The analysis of the proteomic profiles revealed largely distinct molecular signatures in muscles of tumour‐bearing mice expressing or not RAGE at myofibre level or in the total body, with *Cellular protein metabolic processes* emerging as the only shared term among all groups, highlighting alterations of muscle metabolism as a common hallmark in the presence of cancer. Specifically, enrichment in terms related to cell death and apoptosis characterized muscles of the LLC/*Ager* ^*flox*^ mice that survived at 25 dpi, suggesting a condition of late‐stage CC in these animals. Hallmarks of apoptotic cell death, including DNA fragmentation, cleavage of poly (ADP‐ribose) polymerase (PARP) and increased proapoptotic factors, have been reported in skeletal muscles of cachectic animal models and cancer patients [4]. Calpain‐2 (CAPN2), which has a synergistic effect with the UPS in sustaining cancer‐induced muscle proteolysis [37], appeared among the upregulated proteins in LLC/*Ager* ^*flox*^ mice.

Terms related to proteolysis and catabolic processes, and the glycolytic process characterized muscles of LLC/*Ager* ^mKO^ mice. Since proteolysis and catabolism are hallmarks of cancer‐induced muscle wasting, these results suggest that CC is ongoing in these animals. However, an intensified glycolytic process appears in contrast with the myofibre remodeling in favour of MyHC‐I and ‐IIa observed in LLC/*Ager* ^mKO^ muscles, since these fibres are typically associated with a more oxidative metabolism. Although the balance between oxidative and glycolytic metabolisms was found to be maintained in muscles of cachectic cancer patients, a decreased glycolysis associated with decreased oxidative activity has been reported in skeletal muscles of CC mouse models [22]. Our findings suggest that, besides promoting a myofibre remodeling, skeletal muscles of LLC/*Ager* ^mKO^ mice try to counteract cancer‐induced wasting by improving the glycolytic process in the absence of mitochondrial deficits through a Warburg‐like effect [22]. An increased expression of glycolytic enzymes in slow myofibres has been observed in other conditions. Murgia and coworkers reported an increase of enzymes involved in carbohydrate metabolism in slow myofibres during ageing, which is likely to increase the availability of carbon intermediates and sustain protein synthesis [38]. Indeed, although glycolysis yields less ATP than oxidative phosphorylation, it provides metabolic intermediates that can be redirected into the biosynthesis of nucleotides, amino acids and fatty acids, thereby supporting muscle growth [39]. Thus, we can speculate that the glycolytic reprogramming observed in LLC/*Ager* ^mKO^ muscles may represent an adaptive metabolic strategy aimed at preserving muscle integrity in cancer conditions. The potential role of RAGE in modulating muscle metabolism and supporting systemic energy homeostasis is intriguing and deserves further investigation.

In LLC/*Ager* ^mKO^ muscles, we also observed an increased expression of PGC‐1α, which generally shifts metabolism away from glycolysis towards oxidative phosphorylation and fatty acid oxidation by promoting mitochondrial biogenesis. However, in specific contexts characterized by a high‐energy demand, the upregulation of PGC‐1α might be the result of improved glycolysis, for example, through the production of ROS induced by lactate, a by‐product of the glycolysis itself [40]. The observed upregulation of antioxidant enzymes is likely to be the consequence of this ROS generation. PGC‐1α, in turn, can lead to the upregulation of genes typical of oxidative myofibres [41], thus conferring resistance to cancer‐induced muscle atrophy.

Terms related to catabolic processes, blood coagulation, fibrinolysis and immune response characterized the proteomic profile of LLC/*Ager* ^−/−^ mice, indicating that muscle wasting was present to some extent, even if these animals showed no significant loss of muscle mass at 25 dpi. In previous work, we reported that LLC/*Ager* ^−/−^ mice started losing body weight significantly from 30 dpi, and an ~50% decrease in muscle weight was observed in the surviving mice at 40 dpi [10]. The appearance of terms related to the immune response only in these animals leads us to speculate that in the total absence of RAGE, a peculiar immune response occurs in tumour‐bearing mice, culminating in reduced systemic and muscle inflammation.

The proteomic profiles of the three animal models used reflected their Kaplan–Meier curves, in which LLC/*Ager* ^*flox*^ mice showed the lowest survival rate, followed by LLC/*Ager* ^mKO^ and LLC/*Ager* ^−/−^ mice, further supporting that different cachectic stages characterize these tumour‐bearing animals in dependence on RAGE expression and activity.

Systemic inflammation plays a fundamental role in the development of CC, and several studies demonstrate that inflammation is sufficient to drive muscle wasting in different pathological conditions. In CC, cytokines released by both the cancer cells and immune cells activated by the tumour presence trigger downstream signaling pathways culminating in muscle catabolism [2, 3]. Blockade of cytokines such as TNF‐α and IL‐6, which mainly orchestrate the inflammatory response, translates into reduced loss of body and muscle weights in preclinical models of CC, including LLC‐bearing mice [42]. However, whether systemic inflammation exerts a direct effect on muscle tissue or this effect is mediated/exacerbated by other tissues or factors remains to be established. We reported that the total ablation of RAGE in mice translates into restrained systemic inflammation in cancer conditions [10]. Here, we observe a reduction of cytokines known to sustain CC (i.e., TNF‐α, IL‐1β, IL‐4, IL‐6 and LIF) in muscles and/or serum of tumour‐bearing mice lacking RAGE only at muscle level, indicating that, once re‐expressed in myofibres, muscular RAGE has a major role in driving the inflammatory state. The concept of skeletal muscle tissue as an immune organ able to release a variety of CCFs is supported by a recent study on cachectic cancer patients in which some muscle‐derived myokines appeared to contribute to the pathogenesis of CC and to be well integrated into the regulatory network of the factors responsible for the cachectic condition in these patients [43].

RAGE expressed in immune cells certainly has a role in sustaining CC, especially the inflammatory component, as evidenced by the higher resistance to muscle wasting and higher survival shown by *Ager* ^−/−^ compared with *Ager* ^mKO^ mice after injection with tumour cells. The specific role of immune RAGE in CC needs to be investigated in the near future.

The novel skeletal muscle‐conditional experimental model generated in this work (*Ager* ^mKO^ mouse) appears as a useful tool for investigating the role of RAGE re‐expressed at myofibre level in inducible diseases involving muscle tissue or in the cross‐talks established by muscles with other organs (e.g., through myokine release) in conditions characterized by the re‐expression of RAGE in myofibres.

Although CC is associated with morbidity and mortality, it remains a scarcely understood syndrome due to its highly complex multifactorial nature, and efficacious treatments are lacking [44]. Our study demonstrates that RAGE engagement at the myofibre level is a determinant for muscle wasting and inflammation in cancer conditions, even though the total ablation of RAGE translates into the highest protection against CC. The finding that RAGE amounts are increased in muscles of cancer patients even in the pre‐cachectic stage indicates that the re‐expression of RAGE in muscles is an early event in CC and highlights the role of muscular RAGE in promoting and sustaining CC. Interestingly, muscles of cancer patients show alteration of the same pathway affected by RAGE in the experimental model of CC investigated, supporting the existence of a common RAGE‐centred mechanism.

Thus, the molecular targeting of RAGE might represent a promising approach to counteract the onset and progression of the cachectic syndrome and prolong patients' survival. Several RAGE inhibitors have been tested in preclinical models of diseases [45], with some of them being at various phases of clinical trials [46]. In particular, azeliragon (also known as TTP488 or PF‐04494700) is an oral small molecule that has been used in combination with temozolomide and radiotherapy in patients with newly diagnosed glioblastoma in a clinical trial, which reported no toxicities even at the maximum dose of azeliragon used (20 mg/day) [47]. The fact that RAGE is poorly expressed in most organs in basal conditions further supports the pharmacological inhibition of this receptor as a treatment for cachexia in cancer patients.

## Ethics Statement

We certify that we comply with the ethical guidelines for authorship and publishing.

## Conflicts of Interest

The authors declare no conflicts of interest.

## Supporting information


**Table S1:** List of reagents and resources.
**Table S2:**, related to Figure 5. ShinyGO analysis of LLC/*Ager* ^*flox*^, LLC/*Ager* ^−/−^ and LLC/*Ager* ^mKO^ mice vs. internal controls.
**Figure S1:**, related to Figure 1. Characterization of *Ager* ^mKO^ mice. (A) Schematic representation of the experimental protocol. (B,C) Body (B) and muscle (C) weights of 3‐ and 6‐month‐old *Ager* ^*flox*^ and *Ager* ^mKO^ mice (*n* = 8) as evaluated 30 days after treatment with tamoxifen. TA, *tibialis anterior*; GC, gastrocnemius; QF, *quadriceps femoris*. (D) Muscle functionality of 3‐month‐old *Ager* ^*flox*^ and *Ager* ^mKO^ mice (*n* = 8) as evaluated by Kondziela's inverted screen test before (T0) and 30 days after (T30) treatment with tamoxifen. Each point represents an individual mouse. Data are mean ± SEM. Student's *t*‐test; no statistical significances were found.
**Figure S2:**, related to Figures 1 and 2. Total protein staining. (A,B) Total proteins of blots used for the detection of RAGE (A; related to Figure 1F) or PGC‐1α (B; related to Figure 2F) were visualized by No‐Stain Protein Labeling reagent.
**Figure S3:**, related to Figure 1. Evaluation of LLC tumour masses developed in the mouse models. (A) Weights of LLC tumour masses excised from *Ager* ^*flox*^, *Ager* ^mKO^ and *Ager* ^−/−^ mice at 25 dpi. (B,C) Representative images of H&E staining of formalin‐fixed paraffin‐embedded LLC tumour masses (B). The percentages of necrotic areas were determined (C). Bars (B), 200 μm. Data are mean ± SEM. One‐way ANOVA; no statistical significances were found.
**Figure S4:**, related to Figure 2. Effects of RAGE ablation in muscles on cancer‐induced muscle wasting. (A) Representative images of H&E staining of *tibialis anterior* (TA) muscles of *Ager* ^*flox*^ and *Ager* ^mKO^ mice in the absence (Ctrl) or presence of injected LLC cells. Bars, 100 μm. (B) Distribution 14 of cross‐sectional areas (CSAs) of TA muscles of *Ager* ^*flox*^, *Ager* ^mKO^ and *Ager* ^−/−^ mice injected or not with LLC cells. (C) Percentage changes of CSAs in TA muscles of LLC/*Ager* ^*flox*^, LLC/*Ager* ^mKO^ and LLC/*Ager* ^−/−^ mice vs. internal control. (D) Representative western blot images of MyHC‐II, and total and phosphorylated Akt and GSK‐3β, in TA muscles of *Ager* ^*flox*^ and *Ager* ^mKO^ mice injected or not with LLC cells at 25 dpi (*upper panel*). Reported are the relative densities with respect to the total form or α‐actinin (*lower panel*). (E) Real‐time PCR for *Fbxo32* and *Trim63* in TA muscles of *Ager* ^*flox*^ and *Ager* ^mKO^ mice injected or not with LLC cells at 25 dpi. *Tbp* was used as a housekeeping gene. Data are mean ± SEM. One‐way ANOVA; *p < 0.01, **p < 0.01, ***p < 0.001, ****p < 0.0001; ##*p* < 0.01 vs. internal Ctrl.
**Figure S5:**, related to Figure 2. Cancer‐induced fat loss in dependence on RAGE expression. Weights of inguinal (iWAT) and epididymal (eWAT) adipose tissues excised at 25 dpi from *Ager* ^*flox*^ and *Ager* ^mKO^ mice injected (*n* = 8) or not (Ctrl; *n* = 6) with LLC cells. Data are mean ± SEM. One‐way ANOVA; *p < 0.01, **p < 0.01, ***p < 0.001 vs. internal Ctrl.
**Figure S6:**, related to Figure 4. Expression of Warburg effect‐related enzymes is increased in LLC/*Ager* ^mKO^ mice. (A) Reported are the mass spectrometry (MS) intensities of the enzymes related to glycolysis and Warburg effect that emerged as modulated from the proteomic analysis of GC muscles of LLC/*Ager* ^mKO^ vs. Ctrl/*Ager* ^mKO^ mice at 25 dpi. The MS intensities of *Ager* ^*flox*^ and *Ager* ^−/−^ 16 muscles are reported for comparison. (B) Western blotting analysis of ALDOA, LDHA, PGK1 and PKM1/2 in *Ager* ^*flox*^ and *Ager* ^mKO^ mice in the absence (Ctrl) or presence (LLC) of tumour cells (*left panel*). A representative blot of total protein is reported. The relative quantities normalized to total proteins were determined (*right panel*). *p < 0.01, **p < 0.01, statistically significant vs. internal Ctrl. ALDOA, fructose‐bisphosphate aldolase A; ENO1, enolase 1; ENO3, enolase 3; GAPDH, glyceraldehyde‐3‐phosphate dehydrogenase; HK1, hexokinase‐1; LDHA, lactate dehydrogenase A; PGK1, phosphoglycerate kinase 1; PKM, pyruvate kinase; TPI1, triosephosphate isomerase 1.
**Figure S7:**, related to Figure 5. (A,B) Total protein staining of blots used for the detection of MyHCII and RAGE (A; related to Figure 5A), or PGC‐1α (B; related to Figure 5B) in sample biopsies of *rectus abdominis* muscles of pre‐cachectic (*n* = 4) or cachectic (*n* = 6) cancer patients, and control subjects (Ctrl; *n* = 3), as visualized by No‐Stain Protein Labeling reagent.
