# Supplementary material for: RAGE Re‐Expressed at Myofibre Level Drives Muscle Wasting in Cancer Conditions
Source: J Cachexia Sarcopenia Muscle. 2026 May 9;17(3):e70302. doi: 10.1002/jcsm.70302 (PMC13156705; doi:10.1002/jcsm.70302)
Supplement: Supplementary file 1 — Table S1: List of reagents and resources. Table S2:, related to Figure 5. ShinyGO analysis of LLC/Ager flox , LLC/Ager −/− and LLC/Ager mKO mice vs. internal controls. Figure S1:, related to Figure 1. Characterization of Ager mKO mice. (A) Schematic representation of the experimental protocol. (B,C) Body (B) and muscle (C) weights of 3‐ and 6‐month‐old Ager flox and Ager mKO mice (n = 8) as evaluated 30 days after treatment with tamoxifen. TA, tibialis anterior; GC, gastrocnemius; QF, quadriceps femoris. (D) Muscle functionality of 3‐month‐old Ager flox and Ager mKO mice (n = 8) as evaluated by Kondziela's inverted screen test before (T0) and 30 days after (T30) treatment with tamoxifen. Each point represents an individual mouse. Data are mean ± SEM. Student's t‐test; no statistical significances were found. Figure S2:, related to Figures 1 and 2. Total protein staining. (A,B) Total proteins of blots used for the detection of RAGE (A; related to Figure 1F) or PGC‐1α (B; related to Figure 2F) were visualized by No‐Stain Protein Labeling reagent. Figure S3:, related to Figure 1. Evaluation of LLC tumour masses developed in the mouse models. (A) Weights of LLC tumour masses excised from Ager flox , Ager mKO and Ager −/− mice at 25 dpi. (B,C) Representative images of H&E staining of formalin‐fixed paraffin‐embedded LLC tumour masses (B). The percentages of necrotic areas were determined (C). Bars (B), 200 μm. Data are mean ± SEM. One‐way ANOVA; no statistical significances were found. Figure S4:, related to Figure 2. Effects of RAGE ablation in muscles on cancer‐induced muscle wasting. (A) Representative images of H&E staining of tibialis anterior (TA) muscles of Ager flox and Ager mKO mice in the absence (Ctrl) or presence of injected LLC cells. Bars, 100 μm. (B) Distribution 14 of cross‐sectional areas (CSAs) of TA muscles of Ager flox , Ager mKO and Ager −/− mice injected or not with LLC cells. (C) Percentage changes of CSAs in TA muscles of LLC/Ager flox , LLC/Ager [file JCSM-17-e70302-s001.pdf]

SUPPLEMENTARY MATERIAL

Table S1. List of reagents and resources.

REAGENT or RESOURCE	SOURCE	IDENTIFIER
**Antibodies**		
Primary antibodies		
Goat polyclonal anti-RAGE (N16)	Santa Cruz Biotech.	Cat#sc-8230
Mouse monoclonal anti-MyHC-II (MF20)	eBiosciences	Cat#14-6503
Mouse monoclonal anti-MyHC-IIa (SC-71)	DSHB	ID: AB_2314830
Mouse monoclonal anti-MyHC-IIb (BF-F3)	DSHB	ID: AB_2266724
Mouse monoclonal anti-MyHC-IIx (6H1)	DSHB	ID: AB_2314830
Mouse monoclonal anti-Myosin (Skeletal, Slow) (NOQ7.5.4.D)	Sigma-Aldrich	Cat#M8421
Mouse monoclonal anti-phospho-GSK-3β (F-2)	Santa Cruz Biotech.	Cat#sc-373800
Mouse monoclonal anti-α-Actinin (H-2)	Santa Cruz Biotech.	Cat#sc-17829
Mouse monoclonal GSK-3β (E-11)	Santa Cruz Biotech.	Cat#sc-377213
Rabbit monoclonal anti-Akt (pan) (C67E7)	Cell Signaling Tech.	Cat#4691
Rabbit monoclonal anti-Aldolase A (D73H4)	Cell Signaling Tech.	Cat#8060
Rabbit monoclonal anti-LDHA (C4B5)	Cell Signaling Tech.	Cat#3582
Rabbit monoclonal anti-Myosin (Skeletal, Slow) (EPR22697-17)	Abcam	Cat#ab234431
Rabbit monoclonal anti-PGK1 (E9R7O)	Cell Signaling Tech.	Cat#63536
Rabbit monoclonal anti-phospho-Akt (Ser473) (D9E)	Cell Signaling Tech.	Cat#4060
Rabbit monoclonal anti-PKM1/2 (C103A3)	Cell Signaling Tech.	Cat#3190
Rabbit polyclonal anti-PGC1α	ABclonal	Cat#A12348
Secondary antibodies		
Goat anti-mouse IgG/IgM-HRP conjugated	Merck	Cat#AP130P
Goat anti-Mouse IgG1, Alexa Fluor™ 488	Invitrogen	Cat#A-21121
Goat anti-Mouse IgM (Heavy chain), Alexa Fluor™ 555	Invitrogen	Cat#A-21426
Goat anti-rabbit IgG-HRP conjugated	Sigma-Aldrich	Cat#A9169
Goat anti-Rabbit IgG (H+L) Cross-Adsorbed, DyLight™ 405	Invitrogen	Cat#35550
Horse anti-Goat IgG Biotinylated	Vector Laboratories	Cat#BA-9500
Rabbit anti-goat IgG-HRP conjugated	Sigma-Aldrich	Cat#A8919
**Chemicals, Peptides, and Recombinant Proteins**		
Acrylamide bis-solution, 40% W/V	Serva	Cat#10681.01
Agarose	Aurogene	Cat#AU1103
Aprotinin from bovine lung	SERVA	Cat#13718.03
Blotto, non-fat dried milk	Santa Cruz Biotech.	Cat#sc-2325
Bovine Serum Albumin (BSA)	Sigma-Aldrich	Cat#A7030-100G
Corn Oil	Sigma-Aldrich	Cat#C-8267
DAPI dihydrochloride	Sigma-Aldrich	Cat#D 9542
Diaminobenzidine tetrahydrochloride (DAB)	Sigma-Aldrich	Cat#D-5637

Dithiothreitol (DTT)	SERVA	Cat#20710.04
Eosin Y, 1%	Bio Optica	Cat#05-1007/L
Eukitt® mountant	Bio Optica	Cat#09-00500
Formaldehyde	Bio Optica	Cat#05-01005Q
Gelatin solution	Sigma-Aldrich	Cat#G1393
Glycine	Serva	Cat#23391.03
Iodoacetamide	Sigma-Aldrich	Cat#I6125
Leupeptin	SERVA	Cat#51867.03
Mayer’s Hematoxylin	Bio Optica	Cat#05-06002/L
Novel Juice	BIO-HELIX	Cat#LD001-1000
Paraformaldehyde (PFA)	Sigma-Aldrich	Cat#158127-5G
Pepstatin	SERVA	Cat#52682.03
Phenylmethylsulfonyl fluoride (PMSF)	SERVA	Cat#32395.03
Phosphate-buffered saline (PBS)	Euroclone	Cat#ECB4004L
ProLong glass antifade mountant	Thermo Fisher Scientific	Cat#P36982
RIPA Buffer	Thermo Scientific™	Cat#89900
Sodium dodecyl sulfate (SDS)	PanReach AppliChem	Cat#A0675
Tamoxifen	Sigma-Aldrich	Cat#T-5648
Tris(idrossimetil)amminometano (TRIS)	SERVA	Cat#37186.02
TRIsure™	Bioline	Cat#BIO-38033
Triton® X-100	Serva	Cat#39795.02
Trypsin (2.5%)	Gibco	Cat#15090-046
**Critical Commercial Assays**		
PowerUp SYBR Green Master Mix	Applied Biosystems	Cat#A25742
Mouse Magnetic ProcartaPlex Serum/Plasma MAGPIX Liquid	Thermo Fisher Scientific	Cat#PPX-08
MyTAQ extract-PCR kit	Meridian	Cat#BIO-21126
MyTAQ HS mix	Meridian	BIO-25045
PrimeScript™ RT reagent Kit with gDNA Eraser	Takara	Cat#RR047B
PureDireX Genomic DNA Isolation kit	PureDireX	PDC11-0100
VECTASTAIN® Elite® ABC HRP Kit (Peroxidase, Standard)	Vector Laboratories	Cat#PK-6100
Western Bright Quantum HRP substrate	Advansta	Cat#K-12042-D20
**Experimental Models: Cell Lines**		
Lewis lung carcinoma (LLC) cells	ATCC, American Type Culture Collection	Cat#CRL-1642
**Culture medium**		
Dulbecco's Modified Eagle's Medium (DMEM)	Gibco	Cat#41966-029
Fetal bovine serum (FBS)	Gibco	Cat#10270-106
Penicillin-Streptomycin solution	Euroclone	Cat#ECB30010
**Experimental Models: Organisms/Strains**		
C57BL/6 *Ager*$^{-/-}$ mice	MyoLab, Perugia (Italy)	N/A
C57BL/6 *Ager*^{floxx} mice	Provided by Schmidt Ann Marie, USA	N/A

C57BL/6 *Ager*^{mKO} mice	MyoLab, Perugia (Italy)	N/A
C57BL/6 HSA-MCM mice	Jackson	Cat# IMSR_JAX:025750
**Oligonucleotides**		
Primers for PCR		
Ager (Flox sequence) *Fw*-AATGACAGGCAGAGGGCTAA; *Rv*-CCTGGTGGGAAAGAAGACAG.	Invitrogen	N/A
Ager (to detect deleted or not-deleted gene) *Fw*-CCCTCCCACAATGATGCTAT; *Rv*-CCTGGTGGGAAAGAAGACAG.	Invitrogen	N/A
Cre *Fw*-CAGGTAGGGCAGGAGTTGG (hACTA1); *Rv*-TTTGCCCCCTCCATATAACA (rabbit β-globin intron II)	Invitrogen	N/A
Primers for real-time PCR		
mAger *Fw*-CACTTGTGCTAAGCTGTAAGGG; *Rv*-CATCGACAATTCCAGTGGCTG	Invitrogen	N/A
mFbxo32 *Fw*-TCAGCAGCCTGAACTACGAC; *Rv*-GCGCTCCTTCGTACTTCCTT	Invitrogen	N/A
mIl1b *Fw*-TGACGTTCCCATTAGACAACTG; *Rv*-CCGTCTTTCATTACACAGGACA	Invitrogen	N/A
mIl6 *Fw*-GAACAACGATGATGCACTTG; *Rv*-CTTCATGTACTCCAGGTAGCTATGGT	Invitrogen	N/A
mTbp *Fw-CAAGAACAACAGCCTTCCAC* *Rv-GTGGAGTAAGTCCTGTGCCG*	Invitrogen	N/A
mTnfa *Fw*-ACTGAACTTCGGGGTGATCC; *Rv*-TTGCTACGACGTGGGCTACA	Invitrogen	N/A
mTrim63 *Fw*- ATTGTAGAAGCCTCCAAGGG; *Rv*-GGTGTTCTTCTTTACCCTCTGTG	Invitrogen	N/A
**Instruments**		
Caliper	Exacta Optech	
DMRB epifluorescence microscope equipped with a digital camera	Leica	

FastPrep-24 5G	MP BIOMEDICLAS	N/A
FlexMap 3D® Luminex	Diasorin	
Ibright CL1500 System	Thermo Fisher Scientific	
Miniamp Thermal cycler	Thermo Fisher Scientific	
Nikon Ti-E Inverted Fluorescence Motorized Microscope with Spinning Disc, X-Light V2 LFOV	Nikon	
Olympus BX51 equipped with a digital camera	Olympus	
Orbitrap Exploris 480 mass spectrometer with the High-Field Asymmetric Waveform Ion Mobility Spectrometry System (FAIMS) Pro interface	Thermo Fisher Scientific	N/A
QuantStudio 1 Real-Time PCR system	Applied Biosystems	Cat#A40426
Reversed-phase C18 column	Thermo Fisher Scientific	Cat#174500
UltiMate 3000 RSLCnano system	Thermo Fisher Scientific	N/A
**Software and Algorithms**		
Bioinformatics & Evolutionary Genomics	bioinformatics.psb.ugent.be/webtools/Venn	
Biorender	app.biorender.com	Academic Subscription
GraphPad Prism 10.2.3 (403)	GraphPad Software	Academic Subscription
Ibright analyses software v5.3.0	Thermo Fisher Scientific	
ImageJ software	imagej.nih.gov/ij/	
LAS (Leica Application Suite) software v4.12.0	LEICA	
MetaboAnalyst software 6.0	www.metaboanalyst.ca	
NIS-Elements Advanced Research software	Nikon	
Proteome Discoverer with Chimerys v3.0.0.757	Thermo Fisher Scientific	
QuantStudio Design & Analysis software v1.6.1	Applied Biosystems	
ShinyGO 0.80 enrichment tool	bioinformatics.sdstate.edu/go	
SRplot	www.bioinformatics.com.cn/plot_basic_cluster_heatmap_plot_024_en	
xPONENT® 4.2	Luminex Corporation	

Table S2, related to Figure 5. ShinyGO analysis of LLC/*Ager*^{flox}, LLC/*Ager*$^{-/-}$, and LLC/*Ager*^{mKO} mice *vs* internal controls.

**LLC/*Ager*^{flox} *vs* Ctrl/*Ager*^{flox}**
**Enrichment FDR,"nGenes","Pathway Genes","Fold Enrichment","Pathway","URL","Genes"**
0.0234098718452915,2,3,215.392156862745,"GO:0150173 positive reg. of phosphatidylcholine metabolic proc. "," http://amigo.geneontology.org/amigo/term/GO:0150173"," Capn2 Acsl3"
0.0325796716253066,2,5,129.235294117647,"GO:2001245 reg. of phosphatidylcholine biosynthetic proc. "," http://amigo.geneontology.org/amigo/term/GO:2001245"," Capn2 Acsl3"
0.0341548726577023,2,8,80.7720588235294,"GO:0032071 reg. of endodeoxyribonuclease activity "," http://amigo.geneontology.org/amigo/term/GO:0032071"," Npm1 Hmgb1"
0.0341548726577023,2,8,80.7720588235294,"GO:0034379 very-low-density lipoprotein particle assembly "," http://amigo.geneontology.org/amigo/term/GO:0034379"," Acsl3 Ces1d"
0.0341548726577023,2,8,80.7720588235294,"GO:0050904 diapedesis "," http://amigo.geneontology.org/amigo/term/GO:0050904"," Pecam1 Crkl"
0.0341548726577023,2,8,80.7720588235294,"GO:2000425 reg. of apoptotic cell clearance "," http://amigo.geneontology.org/amigo/term/GO:2000425"," Tgm2 Hmgb1"
0.0341548726577023,3,39,24.8529411764706,"GO:0010765 positive reg. of sodium ion transport "," http://amigo.geneontology.org/amigo/term/GO:0010765"," Kif5b Scn4b Dmd"
0.0341548726577023,6,274,7.07492486045513,"GO:0032412 reg. of ion transmembrane transporter activity "," http://amigo.geneontology.org/amigo/term/GO:0032412"," Scn4b Kif5b Oxsr1 Dmd Hk1 Cfl1"
0.0390633554256966,6,297,6.52703505644682,"GO:0032409 reg. of transporter activity "," http://amigo.geneontology.org/amigo/term/GO:0032409"," Scn4b Kif5b Oxsr1 Dmd Hk1 Cfl1"
0.0341548726577023,9,667,4.35951142076021,"GO:0010942 positive reg. of cell death "," http://amigo.geneontology.org/amigo/term/GO:0010942"," Col18a1 Ctsd Capn2 Eef1a1 Tgm2 Ptpa Tomm40 S100a9 Hmgb1"
0.0325796716253066,11,936,3.79697712418301,"GO:0032880 reg. of protein localization "," http://amigo.geneontology.org/amigo/term/GO:0032880"," Kif5b Ctsd Sumo3 Pecam1 Ppp2r5a Acsl3 Cct2 Crkl Prkaca Npm1 Cfl1"
0.0341548726577023,15,1735,2.79327004577047,"GO:0043933 protein-containing complex organization "," http://amigo.geneontology.org/amigo/term/GO:0043933"," Anp32e Cfl1 Prune1 Tmod1 Acsl3 Cct2 Npm1 H1f0 Kif5b Dmd Ces1d Pecam1 Apcs Tgm2 Hmgb1"
0.0325796716253066,17,1972,2.78524340770791,"GO:0006915 apoptotic proc. "," http://amigo.geneontology.org/amigo/term/GO:0006915"," Anp32e Col18a1 Ctsd Capn2 Aimp1 Nae1 Tgm2 Ptpa 1600014C10Rik S100a9 Npm1 Akr1b3 Crkl Tomm40 Hk1 Hmgb1 Nudt2"
0.00110124180878379,26,3024,2.77787503890445,"GO:0065009 reg. of molecular function "," http://amigo.geneontology.org/amigo/term/GO:0065009"," Il1rap Scn4b Npm1 Kif5b Ctsd Anp32e Sumo3 Psmd6 Apcs Ppp2r5a Cct2 Eef1a1 Ptpa Usp14 Hnrnpc Hmgb1 Ly6c1 H1f0 Crkl Oxsr1 Dmd Pecam1 Hk1 S100a9 Cfl1 Tgm2"
0.0325796716253066,17,2026,2.71100691016782,"GO:0012501 programmed cell death "," http://amigo.geneontology.org/amigo/term/GO:0012501"," Anp32e Col18a1 Ctsd Capn2 Aimp1 Nae1 Tgm2 Ptpa 1600014C10Rik S100a9 Npm1 Akr1b3 Crkl Tomm40 Hk1 Hmgb1 Nudt2"
0.0325796716253066,18,2206,2.63625939949869,"GO:0008219 cell death "," http://amigo.geneontology.org/amigo/term/GO:0008219"," Anp32e Col18a1 Ctsd Capn2 Aimp1 Nae1 Eef1a1 Tgm2 Ptpa 1600014C10Rik S100a9 Npm1 Akr1b3 Crkl Tomm40 Hk1 Hmgb1 Nudt2"
0.0325796716253066,21,2914,2.32836408413743,"GO:0032879 reg. of localization "," http://amigo.geneontology.org/amigo/term/GO:0032879"," Scn4b Col18a1 Aimp1 S100a9 Crkl Kif5b Ctsd Sumo3 Pecam1 Ppp2r5a Acsl3 Cct2 Oxsr1 Tgm2 Prkaca Dmd Npm1 Hk1 Cfl1 Ces1d Hmgb1"
0.0325796716253066,29,4806,1.94955447847054,"GO:0044267 cellular protein metabolic proc. "," http://amigo.geneontology.org/amigo/term/GO:0044267"," Sumo3 Psmc5 Psmd6 Ppp2r5a Nae1 Art3

Spcs2 Eef1a1 Tgm2 Fbl Usp14 S100a9 Eif4a1 Rpl18a Prkaca Pecam1 Cfl1 Npm1 Hmgb1 Crkl Ctsd Mapk12 Capn2 Aimp1 Oxsr1 Ptpa Pcbp2 Dmd Hk1"

LLC/*Ager*^{mKO} *vs* Ctrl/*Ager*^{mKO}

Enrichment FDR,"nGenes","Pathway Genes","Fold Enrichment","Pathway","URL","Genes"
5.25858458588187e-09,8,17,35.5286031938549,"GO:0006735 NADH regeneration "," http://amigo.geneontology.org/amigo/term/GO:0006735"," Aldoa Pkm Hk1 Gapdh Tpi1 Eno3 Pgk1 Eno1b"
5.25858458588187e-09,8,17,35.5286031938549,"GO:0061621 canonical glycolysis "," http://amigo.geneontology.org/amigo/term/GO:0061621"," Aldoa Pkm Hk1 Gapdh Tpi1 Eno3 Pgk1 Eno1b"
5.25858458588187e-09,8,17,35.5286031938549,"GO:0061718 glucose catabolic proc. to pyruvate "," http://amigo.geneontology.org/amigo/term/GO:0061718"," Aldoa Pkm Hk1 Gapdh Tpi1 Eno3 Pgk1 Eno1b"
1.31966978240032e-08,8,19,31.7887502260807,"GO:0061620 glycolytic proc. through glucose-6-phosphate "," http://amigo.geneontology.org/amigo/term/GO:0061620"," Aldoa Pkm Hk1 Gapdh Tpi1 Eno3 Pgk1 Eno1b"
3.23784415128092e-10,10,25,30.1993127147766,"GO:0006007 glucose catabolic proc. "," http://amigo.geneontology.org/amigo/term/GO:0006007"," Aldoa Pkm Hk1 Gapdh Tpi1 Eno3 Pgk1 Actn3 Ldha Eno1b"
5.25858458588187e-09,10,34,22.2053769961593,"GO:0019674 NAD metabolic proc. "," http://amigo.geneontology.org/amigo/term/GO:0019674"," Aldoa Pkm Hk1 Gapdh Tpi1 Eno3 Pgk1 Actn3 Ldha Eno1b"
5.25858458588187e-09,10,35,21.5709376534119,"GO:0019320 hexose catabolic proc. "," http://amigo.geneontology.org/amigo/term/GO:0019320"," Aldoa Pkm Hk1 Gapdh Tpi1 Eno3 Pgk1 Actn3 Ldha Eno1b"
1.40708160416599e-08,10,39,19.3585337915235,"GO:0046365 monosaccharide catabolic proc. "," http://amigo.geneontology.org/amigo/term/GO:0046365"," Aldoa Pkm Hk1 Gapdh Tpi1 Eno3 Pgk1 Actn3 Ldha Eno1b"
4.91350036388408e-09,11,44,18.8745704467354,"GO:0006734 NADH metabolic proc. "," http://amigo.geneontology.org/amigo/term/GO:0006734"," Aldoa Pkm Hk1 Gapdh Vcp Tpi1 Eno3 Pgk1 Actn3 Ldha Eno1b"
3.06072470368967e-12,21,157,10.0984962899731,"GO:0002181 cytoplasmic translation "," http://amigo.geneontology.org/amigo/term/GO:0002181"," Rpl15 Denr Rpl6 Rpsa Eif4h Eif4b Rpl24 Rps15a Rpl19 Rps27a Rps14 Rps6 Rps17 Rpl34 Rpl27 Rpl23 Pkm Eif3e Eif3j2 Eif3k Eef2"
2.1789911012781e-08,38,869,3.30142083763381,"GO:0006518 peptide metabolic proc. "," http://amigo.geneontology.org/amigo/term/GO:0006518"," Npepps Rpl15 Lta4h Eif3e Denr Rps14 Rpl6 Gsta4 Rpsa Eef2 Spcs2 Eif4h Eif4b Rpl24 Rps15a Rpl19 Rps27a Rps6 Rps17 Rpl34 Rpl27 Rpl23 Pkm Eif3k Gapdh Calr Cma1 Glo1 Ncl Aimp1 Lrrc47 Apeh Eif3j2 Rps27 Sod1 Tmed2 Arl6ip5 Otud6b"
5.51222980781345e-09,50,1334,2.82977068166947,"GO:1901565 organonitrogen compound catabolic proc. "," http://amigo.geneontology.org/amigo/term/GO:1901565"," Npepps Psmc3 Rad23a Calr Hmox2 Psmb4 Abhd16a Psmb1 Hspa8 Lta4h Psmd3 Psmb6 Cops3 Hint1 Rps27a Psma6 Psmb5 Cul2 Asrgl1 Ddb1 Psmd1 Psmb7 Nsfl1c Psma7 Rad23b Vcp Lypla2 Psmb2 Psma1 Nedd4 Psma4 Skp1 Trim72 Psma3 Psma5 Psmb3 Pcnp Nt5c2 Ahcyl Cma1 Sec22b Bag3 Vps35 Rdx Kyat3 Rpl23 Bnip3 Psap Cast Csnk2a1"
3.06072470368967e-12,74,2094,2.66803861138189,"GO:1901575 organic substance catabolic proc. "," http://amigo.geneontology.org/amigo/term/GO:1901575"," Hk2 Npepps Psmc3 Rad23a Calr Hmox2 Psmb4 Abhd16a Psmb1 Hspa8 Lta4h Psmd3 Psmb6 Cops3 Hint1 Rps27a Psma6 Psmb5 Tpi1 Cul2 Asrgl1 Ddb1 Psmd1 Psmb7 Nsfl1c Psma7 Adh5 Rad23b Vcp Lypla2 Psmb2 Aldoa Psma1 Nedd4 Pkm Psma4 Skp1 Hk1 Trim72 Gapdh Eno1b Hnrnpm Psma3 Eno3 Pgk1 Psma5 Psmb3 Pcnp Glo1 Nt5c2

Ahcyl Ywhah Hnrnpab Esd Cma1 Eif3e Sec22b Bag3 Vps35 Rdx Sh3glb1 Kyat3 Nqo2 Stbd1 Rpl23 Bnip3 Psap Actn3 Mtch2 Ldha Cast Prkaa2 Cbr1 Csnk2a1"

2.73391494861494e-09,59,1689,2.63729936378304,"GO:0070727 cellular macromolecule localization "," http://amigo.geneontology.org/amigo/term/GO:0070727"," Cse1l Atad1 Hspa8 Vps26a Rab21 Sar1b Copa Mapre1 Vcp Mapre3 Tmed2 Vps35 Rab3a Vps26b Rdx Spcs2 Gphn Arl1 Tmed5 Rab18 Ywhah Ywhae Timm9 Ywhaz Pkia Txn1 Prkaa2 Ywhag Hk2 Emd Ap2a2 Calr Hdgf Ap1b1 Chp1 Crk Hnrnpab Cct5 Bsg Mtch2 Nsfl1c Exoc4 Bag3 Nedd4 Skp1 Hk1 Sh3glb1 Cltc Tcp1 Csnk2a1 Sorbs2 Psap Lama5 Arl6ip5 Clip1 Arpc2 Pdcd5 Cfl1 Hnrnpm"

5.25858458588187e-09,58,1681,2.60493774160774,"GO:0034613 cellular protein localization "," http://amigo.geneontology.org/amigo/term/GO:0034613"," Cse1l Atad1 Hspa8 Vps26a Rab21 Sar1b Copa Mapre1 Vcp Mapre3 Tmed2 Vps35 Rab3a Vps26b Rdx Spcs2 Gphn Arl1 Tmed5 Rab18 Ywhah Ywhae Timm9 Ywhaz Pkia Txn1 Prkaa2 Ywhag Hk2 Emd Ap2a2 Calr Hdgf Ap1b1 Chp1 Crk Cct5 Bsg Mtch2 Nsfl1c Exoc4 Bag3 Nedd4 Skp1 Hk1 Sh3glb1 Cltc Tcp1 Csnk2a1 Sorbs2 Psap Lama5 Arl6ip5 Clip1 Arpc2 Pdcd5 Cfl1 Hnrnpm"

5.25858458588187e-09,57,1653,2.60338902713592,"GO:0045184 establishment of protein localization "," http://amigo.geneontology.org/amigo/term/GO:0045184"," Cse1l Atad1 Hspa8 Vps26a Rab21 Sar1b Copa Vcp Tmed2 Vps35 Rab3a Vps26b Spcs2 Arl1 Tmed5 Rab18 Ap1b1 Ywhah Ywhae Timm9 Ywhaz Pkia Txn1 Ywhag Hk2 Emd Ykt6 Ap2a2 Calr Chp1 Cct5 Sec22b Exoc4 Arl8b Bag3 Nedd4 Dnm2 Arl6ip5 Hk1 Tom1 Cltc Tcp1 Ppia Psap Mtch2 Rdx Sh3glb1 Clip1 Arpc2 Bsg Psmd9 Pdcd5 Phpt1 Gphn Ttn Cfl1 Hnrnpm"

6.61052039851603e-10,64,1863,2.59360710379187,"GO:0006508 proteolysis "," http://amigo.geneontology.org/amigo/term/GO:0006508"," Npepps Psmc3 Rad23a Calr Psmb4 Psmb1 Psmd3 Psmb6 Cops3 Rps27a Psma6 Cast Psmb5 Ahsg Fetub Cul2 Cndp2 Ddb1 Psmd1 Psmb7 Cops2 Nsfl1c Psma7 Rad23b Vcp Psmb2 Psma1 Nedd4 Psma4 Spcs2 Skp1 Serpinf2 Otud6b Rnpep Trim72 Psma3 Psma5 Psmb3 Pcnp Serpina1e Pm20d2 Cstb Scrn3 Hspa8 Lta4h Hint1 Scrn2 Cma1 Asrgl1 Apeh Pebp1 Arl6ip5 Metap2 Lap3 Pgk1 Pepd Rpl23 Mtch2 Vps35 Pdcd5 Cfl1 Gapdh Eno1b Csnk2a1"

3.06072470368967e-12,82,2477,2.49933754805378,"GO:0009056 catabolic proc. "," http://amigo.geneontology.org/amigo/term/GO:0009056"," Hk2 Npepps Psmc3 Rad23a Calr Hmox2 Psmb4 Abhd16a Psmb1 Hspa8 Lta4h Psmd3 Psmb6 Cops3 Hint1 Rps27a Psma6 Psmb5 Tpi1 Cul2 Asrgl1 Ddb1 Psmd1 Psmb7 Nsfl1c Psma7 Adh5 Rad23b Vcp Lypla2 Psmb2 Aldoa Psma1 Nedd4 Pkm Psma4 Skp1 Hk1 Trim72 Gapdh Eno1b Hnrnpm Psma3 Eno3 Pgk1 Psma5 Psmb3 Pcnp Glo1 Nit1 Nt5c2 Prkaa2 Slc25a4 Ahcyl Ywhah Hnrnpab Esd Cma1 Eif3e Pter Sec22b Atp2a2 Arl8b Bag3 Vps35 Rdx Qsox1 Sh3glb1 Kyat3 Nqo2 Cltc Stbd1 Pepd Rpl23 Bnip3 Psap Actn3 Mtch2 Ldha Cast Cbr1 Csnk2a1"

5.25858458588187e-09,66,2099,2.37393358644028,"GO:0044248 cellular catabolic proc. "," http://amigo.geneontology.org/amigo/term/GO:0044248"," Npepps Psmc3 Rad23a Calr Hmox2 Psmb4 Abhd16a Psmb1 Hspa8 Lta4h Psmd3 Psmb6 Cops3 Hint1 Rps27a Psma6 Psmb5 Tpi1 Cul2 Asrgl1 Ddb1 Psmd1 Psmb7 Nsfl1c Psma7 Adh5 Rad23b Vcp Psmb2 Psma1 Nedd4 Psma4 Skp1 Trim72 Hnrnpm Psma3 Psma5 Psmb3 Pcnp Glo1 Nit1 Nt5c2 Prkaa2 Slc25a4 Ahcyl Hk2 Hnrnpab Esd Eif3e Sec22b Lypla2 Atp2a2 Arl8b Bag3 Qsox1 Sh3glb1 Kyat3 Nqo2 Cltc Stbd1 Rpl23 Bnip3 Psap Vps35 Cbr1 Csnk2a1"

1.73353568495939e-10,119,4806,1.86939149659718,"GO:0044267 cellular protein metabolic proc. "," http://amigo.geneontology.org/amigo/term/GO:0044267"," Psmc3 Rad23a Calr Psmb4 Rpl15 Psmb1 Psmd3 Psmb6 Cops3 Cand1 Rps27a Prkar1a Psma6 Cast Psmb5 Eif3e Ahsg Fetub Denr Ppil1 Cul2 Rps14 Ddb1 Mstn Psmd1 Psmb7 Cops2 Nsfl1c Pkia Psma7 Rad23b Vcp Prkaa2 Lypla2 Psmb2 Padi2 Rpl6 Cand2 Psma1 Hccs Nedd4 Psma4 Rpsa Eef2 Spcs2 Skp1 Phpt1 Serpinf2 Otud6b Eif4h Trim72 Gphn Ppp1r2 Eif4b Psma3 Psma5 Psmb3 Pcnp Ppia Serpina1e Csnk2a1 Rpl24 Rps15a Rpl19 Rps6 Rps17 Rpl34 Rpl27 Rpl23 Pecam1 Ywhae Pkm Cab39 Adprs Pgp Rap2c Eif3k Pm20d2 Cfl1 Gapdh Obscn Adprh Cstb Hspa8 Hint1 Nme2 Ywhaz Sod1 P4hb Ncl Snta1 Pfn2 Aimp1 Txn1 Lrrc47 Mapre3 Vps35 Gnai2 Pebp1 Arl6ip5 Metap2 Phkb Eif3j2 Acp1 Dusp28 Ttn Armt1 Bnip3 Rps27 Psap Mtch2 Adh5 Tmed2 Pbxip1 Chp1 Pdcd5 Hk1 Limch1 Eno1b"

LLC/*Ager*$^{-/-}$ vs Ctrl/*Ager*$^{-/-}$

Enrichment FDR,"nGenes","Pathway Genes","Fold Enrichment","Pathway","URL","Genes"
5.62634460521106e-06,5,18,53.0676328502415,"GO:0072378 blood coagulation fibrin clot formation "," http://amigo.geneontology.org/amigo/term/GO:0072378"," Fn1 Fga Fgb Fgg F2"
7.57120165171138e-07,6,22,52.102766798419,"GO:0042730 fibrinolysis "," http://amigo.geneontology.org/amigo/term/GO:0042730"," Fga Serping1 F2 Fgb Fgg Plg"
1.11625564603516e-05,7,68,19.6662404092072,"GO:0030193 reg. of blood coagulation "," http://amigo.geneontology.org/amigo/term/GO:0030193"," F2 Fga Serping1 Fgb Fgg Plg S100a9"
1.29645985377226e-05,7,70,19.104347826087,"GO:1900046 reg. of hemostasis "," http://amigo.geneontology.org/amigo/term/GO:1900046"," F2 Fga Serping1 Fgb Fgg Plg S100a9"
1.36047932911607e-05,7,71,18.8352725045928,"GO:0050818 reg. of coagulation "," http://amigo.geneontology.org/amigo/term/GO:0050818"," F2 Fga Serping1 Fgb Fgg Plg S100a9"
2.2802082172895e-07,12,183,12.5274411974341,"GO:0007599 hemostasis "," http://amigo.geneontology.org/amigo/term/GO:0007599"," Fn1 F2 Fga Fgb Fgg Serping1 Plg Anxa7 Cfh S100a9 C3 Gnas"
1.40287205475311e-06,11,180,11.6748792270531,"GO:0007596 blood coagulation "," http://amigo.geneontology.org/amigo/term/GO:0007596"," Fn1 F2 Fga Fgb Fgg Serping1 Plg Cfh S100a9 C3 Gnas"
1.43093804839562e-06,11,183,11.4834877643146,"GO:0050817 coagulation "," http://amigo.geneontology.org/amigo/term/GO:0050817"," Fn1 F2 Fga Fgb Fgg Serping1 Plg Cfh S100a9 C3 Gnas"
4.82908324728182e-06,12,269,8.52238564732504,"GO:0006959 humoral immune response "," http://amigo.geneontology.org/amigo/term/GO:0006959"," C3 Apcs Trf S100a9 Cfh Serping1 F2 A2m Fgb Cfb Hpx Fga"
2.58846565735317e-06,20,807,4.73465869295835,"GO:0044257 cellular protein catabolic proc. "," http://amigo.geneontology.org/amigo/term/GO:0044257"," Psmd4 Ube2g2 Psmb6 Cops3 Ube2d1 Derl1 Ddb1 Psmb7 Psma7 Rad23b Psma1 Nedd4 Psma4 Psma3 Psma5 Eloc Rpl5 Bnip3 Mtm1 Plg"
4.82908324728182e-06,19,776,4.67761093679964,"GO:0051603 proteolysis involved in cellular protein catabolic proc. "," http://amigo.geneontology.org/amigo/term/GO:0051603"," Psmd4 Ube2g2 Psmb6 Cops3 Ube2d1 Derl1 Ddb1 Psmb7 Psma7 Rad23b Psma1 Nedd4 Psma4 Psma3 Psma5 Eloc Rpl5 Mtm1 Plg"
1.03115777962586e-05,21,1011,3.96826215972133,"GO:0030163 protein catabolic proc. "," http://amigo.geneontology.org/amigo/term/GO:0030163"," Psmd4 Ube2g2 Psmb6 Cops3 Ube2d1 Derl1 Ddb1 Psmb7 Psma7 Rad23b Psma1 Nedd4 Psma4 Psma3 Psma5 Eloc Ambp Rpl5 Bnip3 Mtm1 Plg"
2.32785031737891e-10,38,1863,3.89675371653948,"GO:0006508 proteolysis "," http://amigo.geneontology.org/amigo/term/GO:0006508"," Psmd4 Ube2g2 Psmb6 Cops3 Ube2d1 Derl1 Ahsg Fetub Serping1 Ddb1 Psmb7 Psma7 Rad23b Pmpcb Psma1 Nedd4 Psma4 Plg Psma3 Psma5 Eloc Ddx3x Aqp1 Blmh C3 Fn1 F2 Ambp A2m Ngp Fgb Fgg Rpl5 Cfb Mtm1 Cfh Fga S100a9"
4.82908324728182e-06,25,1334,3.58027507985138,"GO:1901565 organonitrogen compound catabolic proc. "," http://amigo.geneontology.org/amigo/term/GO:1901565"," Comt Psmd4 Ube2g2 Psmb6 Cops3 Ube2d1 Blmh Derl1 Ddb1 Psmb7 Psma7 Rad23b Psma1 Nedd4 Psma4 Psma3 Psma5 Eloc Ambp Kyat1 Fitm2 Rpl5 Bnip3 Mtm1 Plg"
5.62634460521106e-06,25,1363,3.50409901432263,"GO:0080134 reg. of response to stress "," http://amigo.geneontology.org/amigo/term/GO:0080134"," F2 Fga Macroh2a1 Cops3 Vdac1 Ahsg Serping1 P4hb Txndc12 A2m Fgb Fgg Parp14 Gfer Plg Lgals1 Prmt1 C3 Tmed2 Psma1 Hpx S100a9 Cav3 Rtn4 Cfh"
4.82908324728182e-06,28,1642,3.25774506169571,"GO:0006952 defense response "," http://amigo.geneontology.org/amigo/term/GO:0006952"," Apcs Trf S100a9 Ddx3x Aqp1 Ahsg Serping1 C3 Fn1 Cfh F2 Fga A2m Mylk3 Ngp Fgb Fgg Parp14 Gfer Orm2 Lgals1 Saa1 Bnip3 Cfb Vdac1 Rtn4 Psma1 Hpx"
4.82908324728182e-06,28,1643,3.2557622588584,"GO:0044419 biological proc. involved in interspecies interaction between organis"," http://amigo.geneontology.org/amigo/term/GO:0044419"," Apcs Trf S100a9

Cav2 Ddx3x Aqp1 Serping1 C3 Ddb1 P4hb Fn1 Cfh F2 Fga A2m Nedd4 Fgb Fgg Parp14 Gfer Plg Lgals1 Bnip3 Cfb Kyat1 Saa1 Cyrib Hpx"
1.63485498149749e-09,53,3834,2.64092445170216,"GO:0006950 response to stress "," http://amigo.geneontology.org/amigo/term/GO:0006950"," Aqp1 Ube2g2 Derl1 Ddb1 P4hb Fn1 Apcs F2 Fga Trf Ucp3 Fgb Fgg S100a9 Prkag1 Comt Rtn4 Rad23b Nmnat3 Ddx3x Macroh2a1 Cops3 Vdac1 Ahsg Serping1 C3 Cfh Ufm1 Txndc12 A2m Ryr1 Mylk3 Nedd4 Ngp Parp14 Setd7 Rcsd1 Gfer Plg Orm2 Cav3 Lgals1 Saa1 Bnip3 Cfb Prmt1 Anxa7 Tmed2 Psma1 Hpx Camk2g Gnas Ambp"
5.41687165321241e-06,36,2611,2.63407323530881,"GO:0051246 reg. of protein metabolic proc. "," http://amigo.geneontology.org/amigo/term/GO:0051246"," Ahsg Fetub Serping1 Nedd4 Hhatl Setd7 Ddx3x Aqp1 Macroh2a1 Ube2d1 Rtn4 Derl1 C3 Ddb1 Fn1 Apcs Ppp2r5a F2 Ambp Rad23b A2m Ngp Parp14 Abce1 Rpl5 Psma3 Cav3 Prkag1 Tmed2 Hpx Mtm1 S100a9 Cav2 Ppp5c Gnas Prmt1"
4.84324232704313e-07,55,4806,2.18630697135827,"GO:0044267 cellular protein metabolic proc. "," http://amigo.geneontology.org/amigo/term/GO:0044267"," Psmd4 Ube2g2 Psmb6 Cops3 Ube2d1 Derl1 Ahsg Fetub Serping1 Ddb1 Pias2 Ppp2r5a Psmb7 Psma7 Ufm1 Rad23b Pmpcb Psma1 Nedd4 Psma4 Hhatl Parp14 Phpt1 S100a9 Abce1 Psma3 Prkag1 Psma5 Eloc Prmt1 Rps29 Rpl5 Mylk3 Setd7 Ddx3x Ppp5c Aqp1 Macroh2a1 Camk2g C3 P4hb Fn1 F2 Ambp A2m Mtm1 Ngp Cav3 Bnip3 H1f2 Tmed2 Hpx Cav2 Gnas Plg"

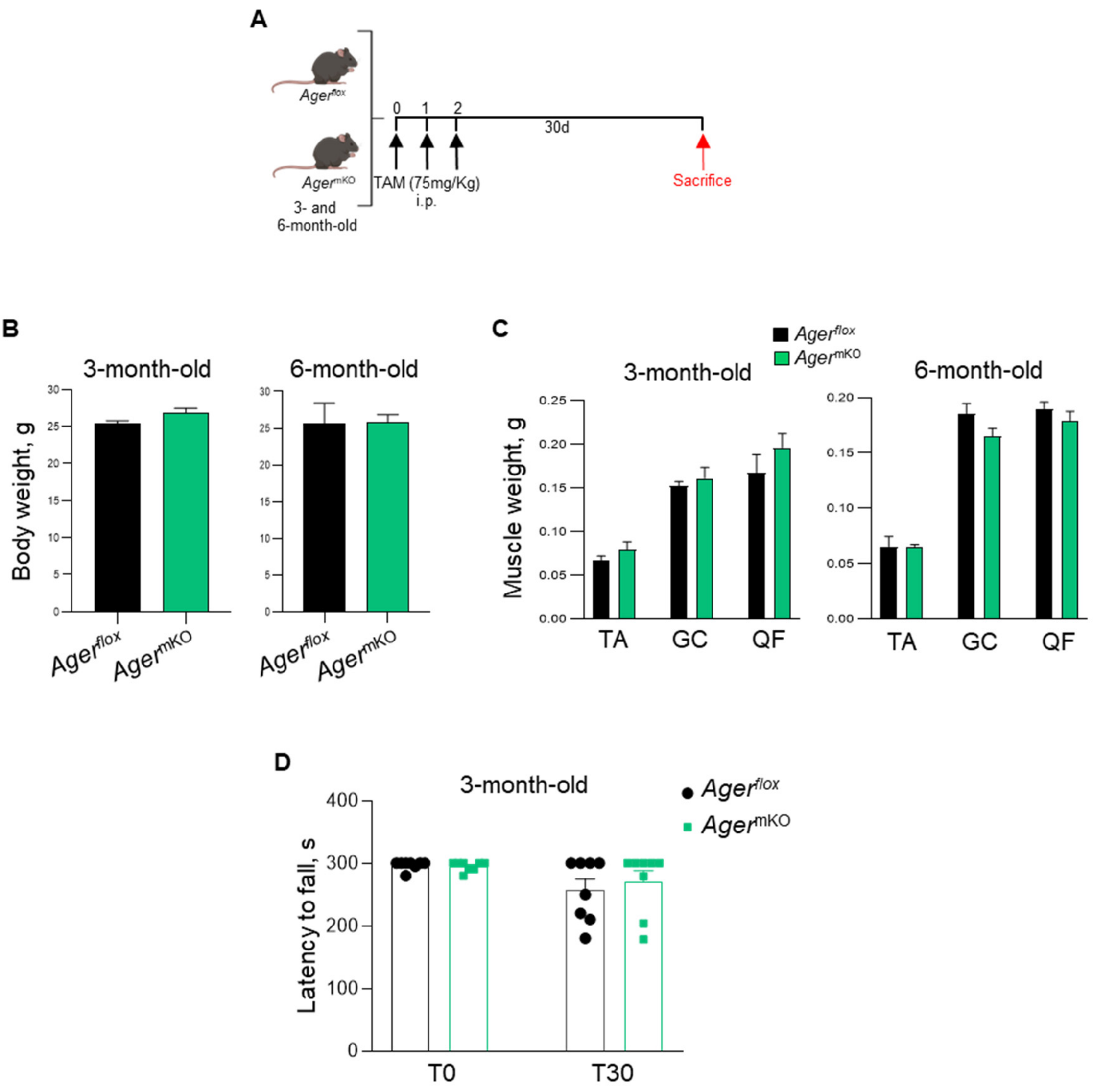

Figure S1, related to Figure 1. Characterization of *Ager*^{mKO} mice. (A) Schematic representation of the experimental protocol. (B,C) Body (B) and muscle (C) weights of 3- and 6-month-old *Ager*^{flox} and *Ager*^{mKO} mice (n=8) as evaluated 30 days after treatment with tamoxifen. TA, *tibialis anterior*; GC, gastrocnemius; QF, *quadriceps femoris*. (D) Muscle functionality of 3-month-old *Ager*^{flox} and *Ager*^{mKO} mice (n=8) as evaluated by Kondziela's inverted screen test before (T0) and 30 days after (T30) treatment with tamoxifen. Each point represents an individual mouse. Data are mean ± SEM. Student's t-test; no statistical significances were found.

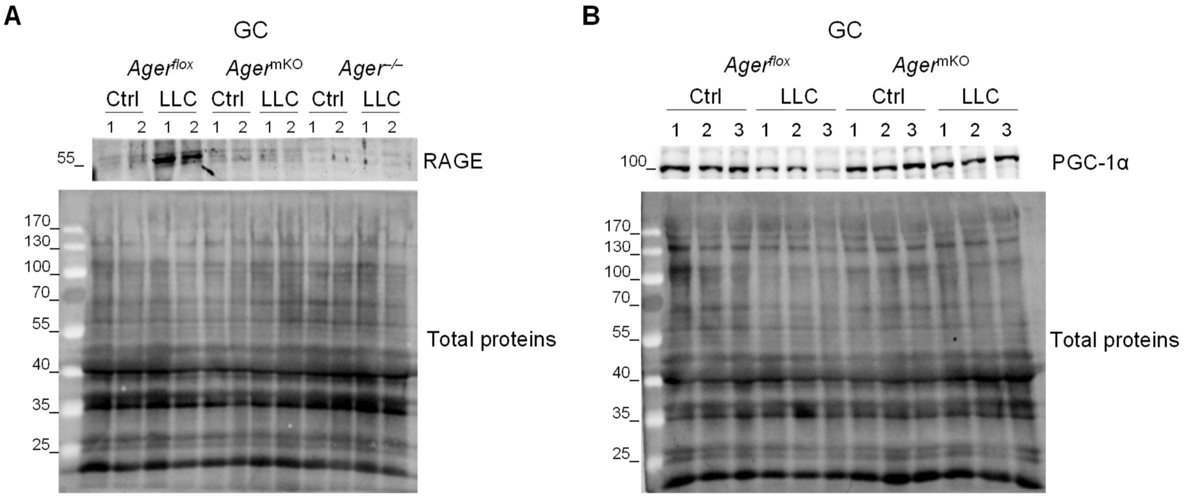

Figure S2, related to Figures 1 and 2. Total protein staining. (A,B) Total proteins of blots used for the detection of RAGE (A; related to Figure 1F) or PGC-1α (B; related to Figure 2F) were visualized by No-Stain Protein Labeling reagent.

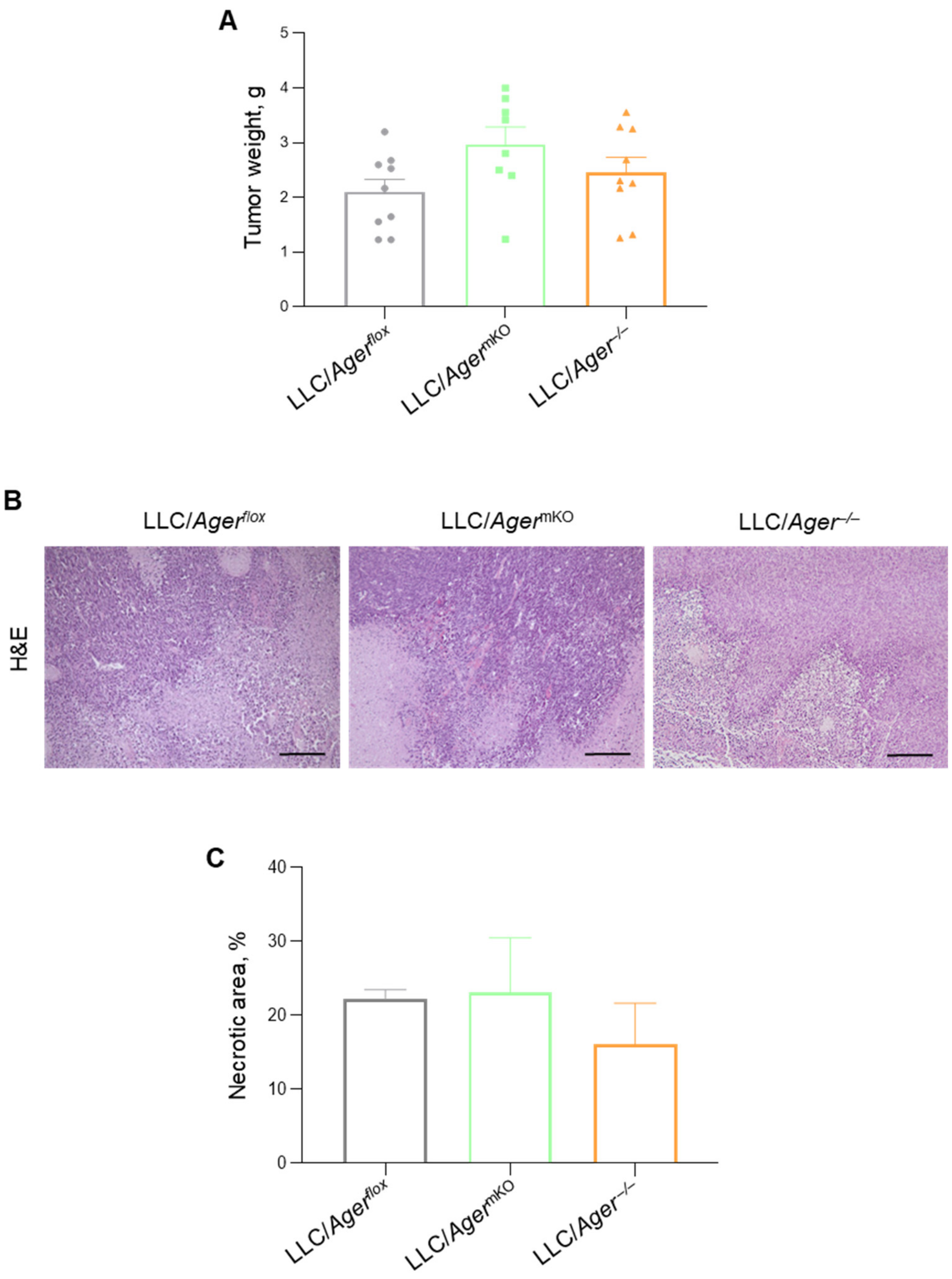

Figure S3, related to Figure 1. Evaluation of LLC tumor masses developed in the mouse models. (A) Weights of LLC tumor masses excised from *Ager*^{flox}, *Ager*^{mKO}, and *Ager*$^{-/-}$ mice at 25 dpi. (B,C) Representative images of H&E staining of formalin-fixed paraffin-embedded LLC tumor masses (B). The percentages of necrotic areas were determined (C). Bars (B), 200 µm. Data are mean ± SEM. One-way ANOVA; no statistical significances were found.

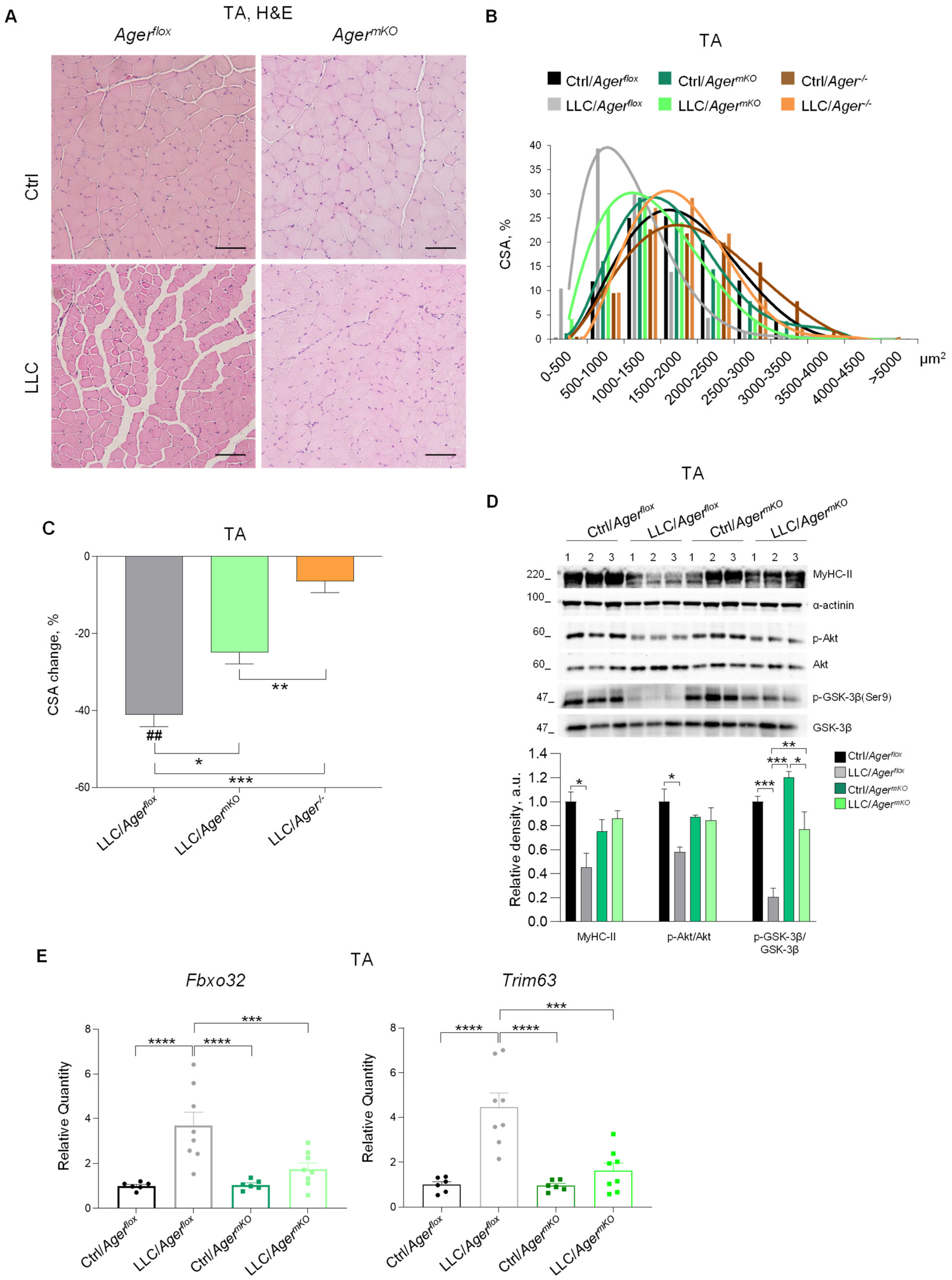

Figure S4, related to Figure 2. Effects of RAGE ablation in muscles on cancer-induced muscle wasting. (A) Representative images of H&E staining of *tibialis anterior* (TA) muscles of *Ager*^{flox} and *Ager*^{mKO} mice in the absence (Ctrl) or presence of injected LLC cells. Bars, 100 µm. (B) Distribution

of cross-sectional areas (CSAs) of TA muscles of *Ager*^{flox}, *Ager*^{mKO}, and *Ager*$^{-/-}$ mice injected or not with LLC cells. (C) Percentage changes of CSAs in TA muscles of LLC/*Ager*^{flox}, LLC/*Ager*^{mKO}, and LLC/*Ager*$^{-/-}$ mice vs internal control. (D) Representative western blot images of MyHC-II, and total and phosphorylated Akt and GSK-3β, in TA muscles of *Ager*^{flox} and *Ager*^{mKO} mice injected or not with LLC cells at 25 dpi (*upper panel*). Reported are the relative densities with respect to the total form or α-actinin (*lower panel*). (E) Real-time PCR for *Fbxo32* and *Trim63* in TA muscles of *Ager*^{flox} and *Ager*^{mKO} mice injected or not with LLC cells at 25 dpi. *Tbp* was used as a housekeeping gene. Data are mean ± SEM. One-way ANOVA; **p* < 0.01, ***p* < 0.01, ****p* < 0.001, *****p* < 0.0001; $^{\#\#}p$ < 0.01 vs internal Ctrl.

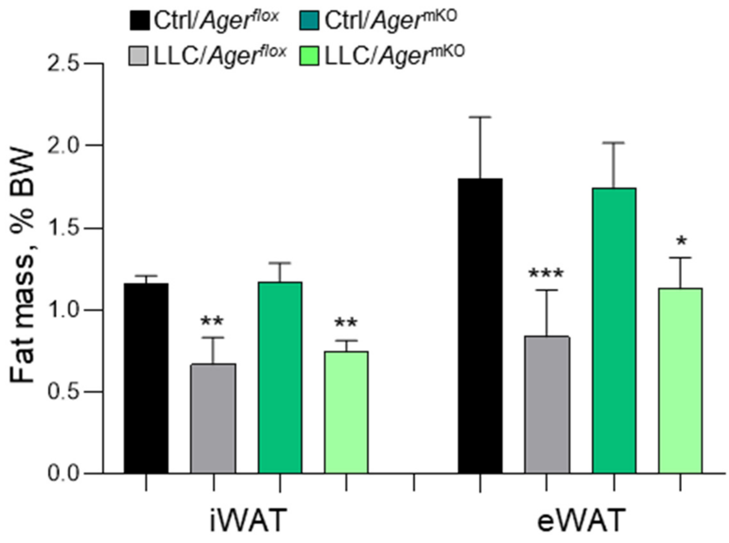

Figure S5, related to Figure 2. Cancer-induced fat loss in dependence on RAGE expression. Weights of inguinal (iWAT) and epididymal (eWAT) adipose tissues excised at 25 dpi from *Ager*^{flox} and *Ager*^{mKO} mice injected (n=8) or not (Ctrl; n=6) with LLC cells. Data are mean ± SEM. One-way ANOVA; **p* < 0.01, ***p* < 0.01, ****p* < 0.001 *vs.* internal Ctrl.

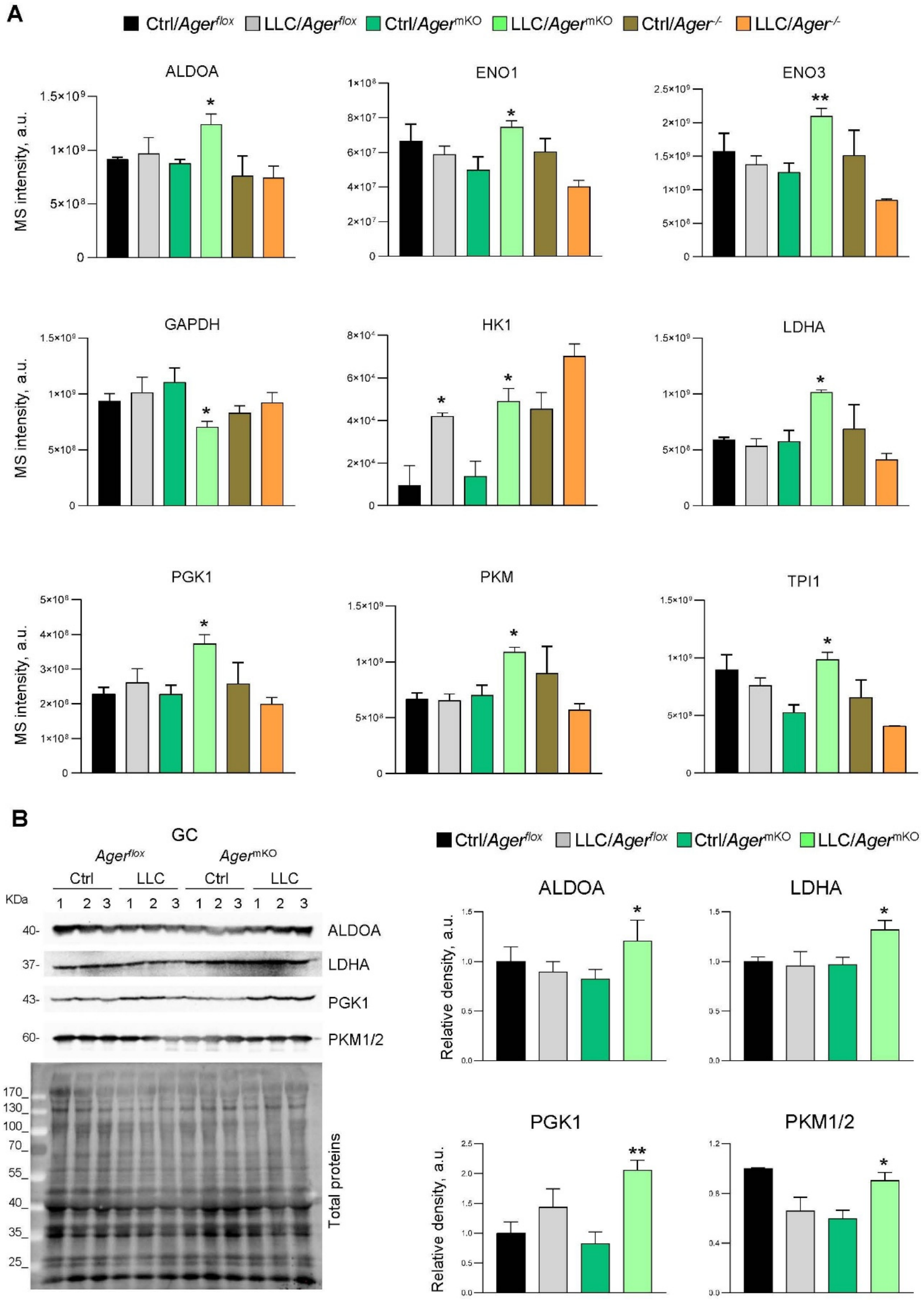

Figure S6, related to Figure 4. Expression of Warburg effect-related enzymes is increased in LLC/*Ager*^{mKO} mice. (A) Reported are the mass spectrometry (MS) intensities of the enzymes related to glycolysis and Warburg effect that emerged as modulated from the proteomic analysis of GC muscles of LLC/*Ager*^{mKO} vs. Ctrl/*Ager*^{mKO} mice at 25 dpi. The MS intensities of *Ager*^{flox} and *Ager*$^{-/-}$

muscles are reported for comparison. (B) Western blotting analysis of ALDOA, LDHA, PGK1, and PKM1/2 in *Ager*^{flox} and *Ager*^{mKO} mice in the absence (Ctrl) or presence (LLC) of tumor cells (*left panel*). A representative blot of total protein is reported. The relative quantities normalized to total proteins were determined (*right panel*). **p* < 0.01, ***p* < 0.01, statistically significant *vs.* internal Ctrl. ALDOA, fructose-bisphosphate aldolase A; ENO1, enolase 1; ENO3, enolase 3; GAPDH, glyceraldehyde-3-phosphate dehydrogenase; HK1, hexokinase-1; LDHA, lactate dehydrogenase A; PGK1, phosphoglycerate kinase 1; PKM, pyruvate kinase; TPI1, triosephosphate isomerase 1.

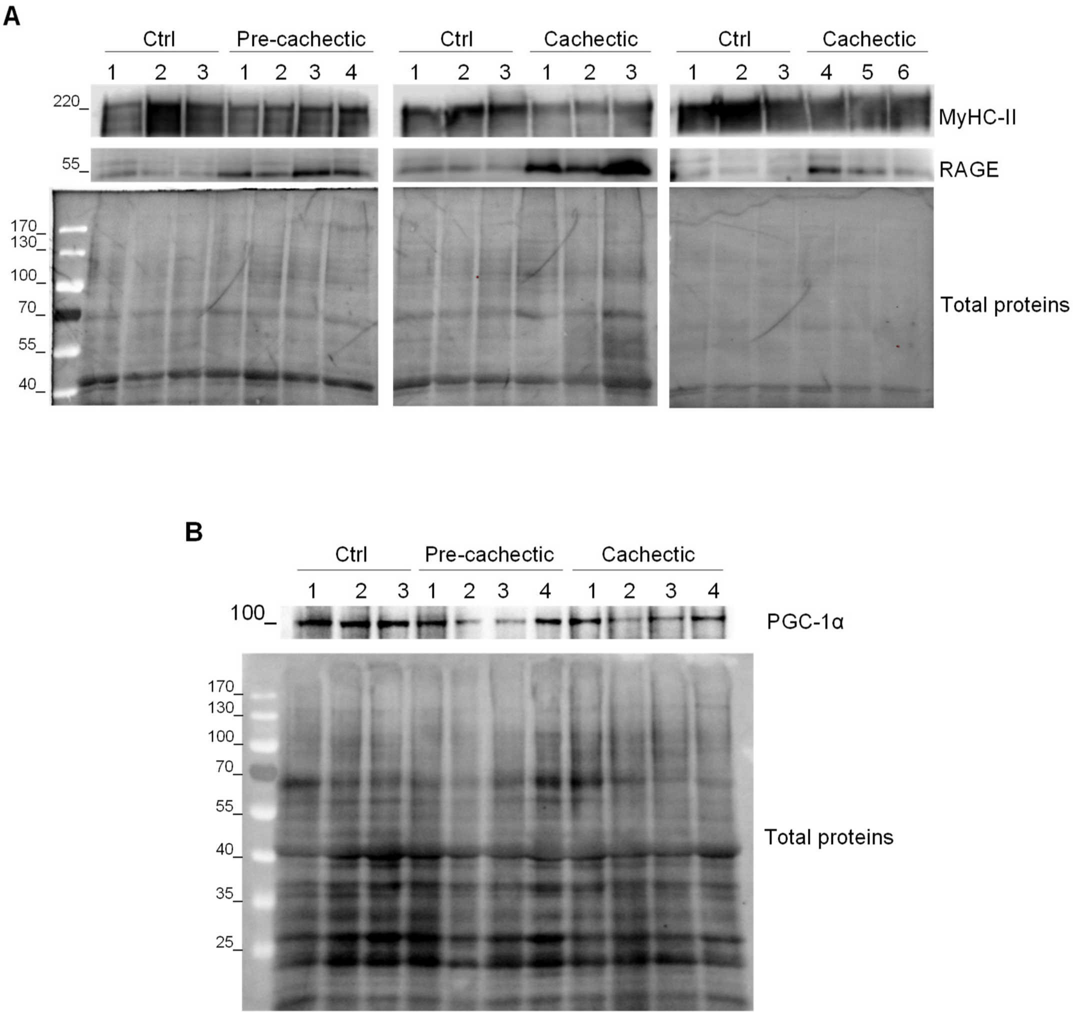

Figure S7, related to Figure 5. (A,B) Total protein staining of blots used for the detection of MyHC-II and RAGE (A; related to Figure 5A), or PGC-1α (B; related to Figure 5B) in sample biopsies of *rectus abdominis* muscles of pre-cachectic (n=4) or cachectic (n=6) cancer patients, and control subjects (Ctrl; n=3), as visualized by No-Stain Protein Labeling reagent.
